# Astrocyte Modulation of Synaptic Plasticity Mediated by Activity-Dependent Sonic Hedgehog Signaling

**DOI:** 10.1523/JNEUROSCI.1336-24.2025

**Published:** 2025-02-03

**Authors:** Anh Duc Le, Marissa Fu, Ashley Carper, Elizabeth Zegarowicz, Riya Kumar, Gloria Zacharias, A. Denise R. Garcia

**Affiliations:** ^1^ Departments of Biology, Drexel University, Philadelphia, Pennsylvania 19104; ^2^ Neurobiology and Anatomy, Drexel University College of Medicine, Philadelphia, Pennsylvania 19129

**Keywords:** astrocyte, Hevin, plasticity, Sonic hedgehog, SPARC, synapse

## Abstract

The influence of neural activity on astrocytes and their reciprocal interactions with neurons has emerged as an important modulator of synapse function. Astrocytes exhibit activity-dependent changes in gene expression, yet the molecular mechanisms by which neural activity is coupled to gene expression are not well understood. The molecular signaling pathway, Sonic hedgehog (Shh), mediates neuron–astrocyte communication and regulates the organization of cortical synapses. Here, we demonstrate that neural activity stimulates Shh signaling in cortical astrocytes and upregulates expression of Hevin and SPARC, astrocyte-derived molecules that modify synapses. Whisker stimulation in both male and female mice promotes activity-dependent Shh signaling selectively in the somatosensory, but not in the visual cortex, whereas sensory deprivation reduces Shh activity, demonstrating bidirectional regulation of the pathway by sensory experience. Selective loss of Shh signaling in astrocytes reduces expression of Hevin and SPARC and occludes activity-dependent synaptic plasticity. Taken together, these data identify Shh signaling as an activity-dependent, molecular signaling pathway that regulates astrocyte gene expression and promotes astrocyte modulation of synaptic plasticity.

## Significance Statement

Understanding how the nervous system orchestrates the complex cellular and molecular interactions that are necessary to adapt to changing environments is a fundamental goal in neuroscience. Neuronal adaption to novel experience is well characterized, however astrocytes are now recognized as key players in modulating synaptic function and plasticity. Like neurons, astrocytes exhibit activity-dependent gene expression. However, the mechanisms by which activity is coupled to gene expression are poorly defined. Here, we show that neural activity stimulates the molecular signaling pathway, Sonic hedgehog (Shh), in astrocytes. Shh signaling promotes expression of synapse-regulating genes and is required for astrocyte modulation of synaptic plasticity. Understanding how astrocytes contribute to synaptic plasticity sheds new light on how experience shapes brain function.

## Introduction

Activity-dependent gene expression is a fundamental property of neurons by which they modify synapses in response to experience. The mechanisms by which neurons convert activity into gene expression are well characterized. Neuronal activation stimulates Ca^2+^ entry into the cell, stimulating downstream molecular signaling programs that lead to activation of immediate-early genes such as *Fos* and *Jun* (Yap and Greenberg, 2018). However, emerging evidence demonstrates that astrocytes also exhibit activity-dependent gene expression (Hasel et al., 2017; Nagai et al., 2019; Sardar et al., 2023). These transcriptional responses are as robust as many classes of neurons (Hrvatin et al., 2018) and include several genes associated with synapse formation and function (Genoud et al., 2006; Allen and Eroglu, 2017; Farhy-Tselnicker et al., 2021). The precise molecular signaling programs that couple synaptic activity to gene expression in astrocytes are not well defined.

The molecular signaling pathway, Sonic hedgehog (Shh), is emerging as an important mediator of reciprocal interactions between neurons and astrocytes. In the postnatal and adult brain, SHH ligand is produced by neurons which acts directly on neighboring astrocytes, regulating SHH-dependent gene expression programs (Garcia et al., 2010; Farmer et al., 2016; Xie et al., 2022). Notably, genetic ablation of pathway activity, selectively in astrocytes, decreases expression of the glial-specific, inward rectifying K^+^ channel, K_ir_4.1, perturbing extracellular K^+^ homeostasis and increasing neuronal excitability (Hill et al., 2019). Conversely, unrestrained Shh signaling promotes synapse formation mediated by increased expression of synaptogenic cues (Xie et al., 2022). These observations point to SHH as a key mediator of neuron–astrocyte interactions that reciprocally shape synapse number and function. Furthermore, noncanonical Shh signaling in neurons is required for establishing corticocortical synapses (Harwell et al., 2012), suggesting diverse and cell-type–specific actions of SHH on synaptic regulation. Here, we focus on Shh signaling in astrocytes mediated by canonical transduction of the pathway.

Transduction of Shh signaling is mediated by transcriptional activation of its effectors, the GLI family of transcription factors, that regulate expression of SHH target genes (Briscoe and Therond, 2013). The binding of SHH to its receptor, Patched (*Ptch1*), relieves inhibition of the obligatory coreceptor, Smoothened (*Smo*), promoting transcription of SHH target genes, including *Gli1*. High levels of SHH promote transcriptional activation of *Gli1*, which acts as a reliable readout of pathway activation (Bai et al., 2002). At baseline, the transcriptional abundance of *Gli1* in the cortex is low (Xie et al., 2022), and its expression is localized to astrocytes in layers IV and V, consistent with the distribution of SHH-expressing neurons in layer V (Garcia et al., 2010; Harwell et al., 2012). High-frequency stimulation of cultured hippocampal neurons promotes SHH release that is sensitive to TTX treatment (Su et al., 2017), suggesting that neural activity promotes Shh signaling. Interestingly, SHH protein is localized in axons (Traiffort et al., 1998, 1999; Charytoniuk et al., 2002; Peng et al., 2018), where it is associated with synaptic vesicles (Beug et al., 2011) making it well positioned to couple with neuronal activity. Whether physiological neural activity promotes SHH-mediated interactions between neurons and astrocytes in vivo, and how these interactions influence synapses, is not known.

To examine this, we exposed juvenile mice to an enriched somatosensory environment to stimulate active whisking. We show that sensory and chemogenetic stimulation increases Shh signaling in cortical astrocytes. Exposure to an enriched environment (EE) produced an increase in synapse number and a concomitant increase in expression of Hevin and secreted protein acidic and rich in cysteine (SPARC), matricellular proteins produced by astrocytes that modify synapses. Structural plasticity and experience-dependent upregulation of Hevin and SPARC are occluded in astrocyte-specific conditional knock-out (CKO) mice in which Shh signaling is selectively abolished, demonstrating a requirement for Shh signaling in astrocyte modulation of synapses. Taken together, these findings demonstrate novel, activity-dependent regulation of Shh signaling and establish SHH as a molecular mechanism by which astrocytes couple neuronal activity to gene expression and modulate synaptic plasticity.

## Materials and Methods

### Animals

All experiments were approved by Drexel University's Institutional Animal Care and Use Committee and were conducted according to approved protocols. Transgenic mouse lines are listed in Table 1, used on the C57BL/6 background. Postnatal (P) day 18–90 mice were used for this study. Mice of both sexes were included in each experiment.

**Table 1.** Key resources

Reagent or resource	Source	Identifier
Antibodies		
Polyclonal goat anti-SPARC	R&D Systems	AF942
Polyclonal goat anti-SPARC-like 1 (SPARCL1/Hevin)	R&D Systems	AF2836
Polyclonal chicken anti-β-Gal	Abcam	AB9361
Polyclonal rabbit anti-β-Gal	Invitrogen	A-11132
Polyclonal rabbit anti-c-Fos	Cell Signaling Technology	2250S
Polyclonal rabbit anti-Sox9	Novus	NVP1-85551
Polyclonal rabbit anti-NeuN	Novus	NBP2-10491
Polyclonal sheep anti-CAII	Serotec	AHP206
Polyclonal rat anti-HA	Sigma-Aldrich/Roche	11867423001
Polyclonal rabbit anti-GluA1	Millipore Sigma	AB1504
Polyclonal chicken anti-GFP	Invitrogen	A10262
Mouse strains		
*Gfap-Cre* (line 73.12; Garcia et al., 2004)	The Jackson Laboratory	Stock no. 12886
*Gli1^CreER/+^* (Ahn and Joyner, 2005)	The Jackson Laboratory	Stock no. 7913
*Gli1^nlacZ/+^* (Bai et al., 2002)		
*Shh^CreER/+^* (Harfe et al., 2004)	Generous gift of Dr. Corey Harwell	
Ai14 (B6.Cg Gt(ROSA)26Sor^tm14(CAG-tdTomato)Hze^/J; Madisen et al., 2009)	The Jackson Laboratory	Stock no. 7914
*Smo^fl/fl^* (Long et al., 2001)	The Jackson Laboratory	Stock no. 4526
*Thy1-*GFPm (Feng et al., 2000)	The Jackson Laboratory	Stock no. 7788
Oligonucleotides		
*Hevin*	Invitrogen	
CACTCCGAGTAGTCCCTCCA (forward)		
AAACTGTGAGTGGTGAGCGA (reverse)		
*Sparc*	Invitrogen	
TAGCTGCTCGGAGGGGAATGT (forward)		
TCACCATCCGCATGTTCCCAC (reverse)		
*Gli1*	Invitrogen	
AACATGGCGTCTCAGGGAAG (reverse)		
GACGGAGGTCTCTTTGTCCG (forward)		
*Gpc4*	Invitrogen	
AGTTGCTCGGAAGTGCGACG (forward)		
TGGTCACCGTTGATCTCATAGAGGG (reverse)		
*Gpc6*	Invitrogen	
TAGTCCTGTATTGGCAGCCAC (forward)		
GGCTAATGTCTATAGCAGGGAA (reverse)		
*Thbs1*	Invitrogen	
GAAGCAACAAGTGGTGTCAGT (forward)		
ACAGTCTATGTAGAGTTGAGCCC (reverse)		
*Thbs2*	Invitrogen	
GTAGGTTTTGACGAGTTTGG (forward)		
TCCACATCACCACATAGAAG (reverse)		
*Gapdh*	Invitrogen	
CATGGCCTACATGGCCTCCA (forward)		
TGGGATAGGGCCTCTCTTGCT (reverse)		
Viral constructs		
AAV8-CaMKIIa-hM3D(Gq)-mCherry	Addgene	50476-AAV8
AAV5-hSyn-hM3D(Gq)-HA	Addgene	121539-AAV5
Software		
Prism	GraphPad Software	
ImageJ	Fiji	
Imaris	Oxford Instruments	
CFX Manager	Bio-Rad	

Information regarding mouse strains, antibodies, primer sequences, and other relevant resources used in this study.

### Enriched experience housing

P21 littermates were weaned into either enriched environment (EE) or standard housing (SH) cages for 2 d or 3 weeks. Large rat cages were used. The EE cage contained strings of beads hung from wire lids, positioned densely throughout the cage, requiring mice to constantly interact with the beads. The density and spacing of the beads were altered every 3 d to promote novelty. The SH cage contained only standard hub and nesting materials. A minimum of seven mice was used for each cage. Data from each analysis were collected over 2–5 separate experiments from separate litters.

### Whisker trimming

Whisker trimming procedure was conducted on P21 mice. Mice were anesthetized with isoflurane in an induction chamber (3%) and then placed under dissecting scope with continuous isoflurane inhalation (1.5%). Microscissors were used to trim whiskers on the right side, as close to the skin as possible. Whiskers were trimmed every 3 d for a duration of 3 weeks.

### Tamoxifen administration

Tamoxifen (Sigma-Aldrich) was dissolved in corn oil at a 20 mg/ml concentration at 37°C. For *Gli1^CreER/nlacZ^*;Ai14 mice, three doses of 250 mg/kg tamoxifen were administered by oral gavage over 3 d from P18 to P20, and tissues were analyzed at P42. For *Shh^CreER^*;Ai14 mice, three similar doses were administered over 3 d at the end of the experiment (concurrent with when increases of β-Gal cells were observed). For *Gli1^CreER^*;*Smo^fl/fl^*;Ai14 mice, three similar doses were administered from P18 to P20, and mice were weaned into SH or EE cages from P21 to P23 and analyzed on P23. For morphology analysis of astrocytes, *Gli1^CreER^*;Ai14 mice were administered three doses of tamoxifen from P18 to P20, weaned into SH or EE cages from P21 to P23, and analyzed on P23.

### Virus injection

All AAV injections were conducted on adult mice at P60 or older. Immediately prior to surgery, mice received a subcutaneous injection of carprofen (Rimadyl, 6.8 mg/kg), and a follow-up injection given the day after surgery. Mice were anesthetized with isoflurane in an induction chamber (3%) and then placed in a stereotaxic apparatus (Stoelting) with continuous isoflurane inhalation (1.5%). A midline incision in the scalp was made, and a small area of the skull was removed near the injection site. Stereotaxic coordinates for somatosensory cortex injection were AP −1.5 mm, ML −2.5 mm, and DV −0.5 mm.

Viruses were stored in aliquots at −80°C and kept on ice until injection. Virus titers were ≥3 × 10^12^ vg/ml for AAV8-CaMKIIa-hM3D(Gq)-mCherry and ≥7 × 10^12^ vg/ml for AAV5-hSyn-hM3D(Gq)-HA. Viruses were front-filled into a glass syringe (Hamilton). The injection volume was 1,000 nl, delivered at 200 nl/min controlled by a microinjector (World Precision Instruments). After injection, mice were allowed to recover for 2 weeks before administration of clozapine N-oxide (CNO; Tocris Bioscience). CNO was dissolved in drinking water, aiming to deliver a dosage of 1 mg CNO per kg mouse over 1 week. Control mice received water only.

### Collection of animal tissue

Animals used in experiments were each assigned a unique 4–5-digit ID in order to blind researchers of genotypes and housing conditions. Mice were deeply anesthetized by intraperitoneal injection of ketamine/xylazine/acepromazine, transcardially injected with 100 units of heparin, and perfused with ice-cold PBS followed by 4% paraformaldehyde (PFA; Sigma-Aldrich). Brains were dissected and fixed for at least 4 h in PFA at 4°C and then transferred to 30% sucrose at 4°C for at least 48 h. Brains were cryosectioned (Leica CM3050s) and collected in 40 µm sections. Sections were stored in 0.1 M Tris-buffered saline (TBS) with 0.05% sodium azide at 4°C. Immunohistochemistry was performed with primary antibodies listed in Table 1. Sections were washed three times with 0.1 M TBS at room temperature (10 min/wash) and then blocked in 10% normal serum with 0.5% Triton X-100 (Sigma-Aldrich) for 1 h at room temperature. Sections were then incubated with primary antibody in 0.5% Triton X-100 at 4°C overnight.

### Immunohistochemistry

For fluorescent immunohistochemistry, sections were rinsed three times the following day in 0.1 M TBS and incubated in Alexa Fluor–conjugated secondary antibodies with 10% normal serum in 0.1 M TBS at room temperature for 2 h. Sections were then rinsed in 0.1 M TBS and incubated in DAPI (Life Technologies) for 15 min. Sections were rinsed again in 0.1 M TBS, mounted on microscope slides (Fisherbrand), and coverslipped using ProLong Gold Antifade Mounting Medium (Invitrogen #P10144) and Fisherfinest Premium Cover Glass. For bright-field immunohistochemistry, sections were rinsed as previously described but were incubated with biotinylated secondary antibodies against the primary species (Vector Laboratories) for 1 h. Sections were transferred to avidin–biotin complex solution (Vector Laboratories) for 1 h and visualized using 3,3′-diaminobenzadine (DAB Peroxidase Substrate Kit, Vector Laboratories). Sections were then mounted onto slides as described, dehydrated through increasing concentrations of EtOH, and coverslipped with DPX mounting medium (Thermo Fisher Scientific).

### Fluorescent in situ hybridization (RNAscope)

All fluorescent in situ hybridization (FISH) experiments were performed on brain tissue fixed with 4% paraformaldehyde and processed using cryosectioning at 14 µm and directly slide mounted, with one brain section per slide. The assay was carried out using the “fixed frozen sample preparation and treatment” and the RNAscope Multiplex Fluorescent v2 Assay using the manufacturer's instructions.

Confocal *z*-stack images were acquired using Leica DMI 4000B microscope equipped with TCS SPE confocal system, at 63× for blinded analysis in ImageJ (Fiji). Cells with at least four puncta were identified as positive for *Gli1* mRNA. The number of *Gli1+* cells was divided by the total number of DAPI cells within the *z*-stack and then normalized to SH condition for each layer. For each animal, each layer, at least two *z*-stacks, was analyzed for a minimum of 100 DAPI cells.

*Gli1* puncta were counted using the software QuPath (QuPath 0.5.1 x64). Min–max projections of 63× images were acquired in ImageJ (Fiji) by taking the 10 middle optical sections. These projections were imported into QuPath where regions of interest were drawn around nuclei identified by DAPI, excluding any overlapping cells. The subcellular detection (experimental) analysis was used to automatically detect how many *Gli1* puncta are present. The parameters were set by setting the detection threshold for each channel, as well as defining the “spot and cluster parameters,” making sure to keep consistent between images. The expected spot size was set to 0.5 µm^2^, the minimum spot size was set to 0.3 µm^2^, and the maximum spot size was set to 0.8 µm^2^ with the “include clusters,” “smooth before detection,” “split by intensity,” and “split by shape” boxes all checked. The estimated *Gli1* punctum detection measurement was collected. Cells with more than three puncta were included in the analysis.

### Stereological quantification

The number of cells in each area of the cortex was estimated using a modified optical fractionator and stereological image analysis software (Stereo Investigator, MBF Bioscience) with an upright microscope (Zeiss) with a computer-driven stage. The cortical areas of interest (somatosensory, auditory or visual cortex) was outlined at low magnification, using anatomical landmarks as described in Paxinos and Franklin's *The Mouse Brain in Stereotaxic Coordinates Fourth Edition*. For the somatosensory cortex, sections from A–P bregma 1.33 to −2.15 mm were analyzed. For the visual cortex, sections from A–P bregma −2.27 to −4.59 mm were analyzed. A target cell count of 150 and target coefficient of error (*m* = 1) of <0.1 were used to define the number of sections counted, scan grid, and counting frame size. Counting frames were randomly selected by the image analysis software, and cells were counted at the 40× objective with a differential interference contrast optic filter. Only cells with a clearly labeled cell body were counted.

### Astrocyte morphology analysis

Analysis of morphology was performed in a blinded study design with the Imaris software (Imaris 10.2 for Neuroscientists). Single astrocyte confocal *z*-stack images were acquired at 63×, 2,048 × 2,048 resolution using Leica DMI 4000B microscope equipped with TCS SPE confocal system. Raw images were deconvolved using Leica Stellaris Lightning function. Deconvolved images were reconstructed in Imaris, using the surface function with manual edits to exclude background signal. The volume statistics were taken from the reconstructed surface. Filaments were created from the surface with manual edits to exclude any false processes. The Sholl intersection, branch level, and process length statistics were taken from the reconstructed filaments. Five cells per animal were analyzed, located in the deep layers of the cortex. The mean values of the cells were averaged per animal.

### Fluorescent intensity analysis of Hevin and SPARC

Immunofluorescent slides were imaged using Leica DMI 4000B microscope equipped with TCS SPE confocal system. Regions of interest were identified with a 10× dry objective, and confocal *z*-stacks were acquired with a 40× oil objective. To analyze protein abundance in a single astrocyte, a 20 μm × 20 μm region of interest is drawn in ImageJ around an astrocyte identified by a fluorescent signal of either tdTomato reporter protein or staining for β-Gal or Sox9. This region of interest should contain one astrocyte of interest and not contain signal from other cells. The mean fluorescent intensity is then collected. At least 25 cells were sampled per animal, dispersed throughout multiple sites encompassing the somatosensory cortex. The mean fluorescent intensities of the cells were averaged to produce the fluorescent intensity value for one animal, represented in the graph as one data point. For negative controls, we measured fluorescent signals of Sox9 and found no difference between layers and housing conditions.

### Dendritic spine density analysis

Analysis of spine density was performed in a blinded study design. WT controls were derived from Cre-negative littermates. Leica DMI 4000B microscope equipped with TCS SPE confocal system was used. For analysis in the cortex, apical dendrites of layer V pyramidal neurons located in layers II/III, IV, and V were imaged. For analysis in the hippocampus, apical dendrites from CA1 pyramidal neurons were imaged. Confocal *z*-stacks were collected with 63× oil objective, at 0.35 μm steps. Spine density was determined by first tracing the dendritic segment in ImageJ and obtaining the length. Analyzed segments range from 75 to 125 µm in length. Protrusions were classified into mushroom, intermediate, or filopodial morphologies. Protrusions exhibiting a bright, bulbous head with a large head-to-stalk ratio were classified as mushroom. Thinner protrusions with a dimmer, smaller, or absent head were classified as intermediate. Dim, thin, and elongated protrusions that lack any head were classified as filopodia.

### Fluorescent intensity analysis of GluA1 at dendritic spines and dendritic shaft

GluA1 immunostaining in dendritic spines and dendritic shaft (identified by GFP) was imaged as confocal *z*-stacks, acquired with 63× oil objective, at 0.35 μm steps using Leica DMI 4000B microscope equipped with TCS SPE confocal system. Images were analyzed in ImageJ (Fiji). To measure GluA1 intensity at dendritic spines, an ROI was drawn around the spine head, excluding the spine neck, in a single *z*-plane at its maximal focus (Zhang et al., 2015; Graves et al., 2021). The mean GluA1 signal intensity was measured. For dendritic shaft analysis, 5 μm ROI segments in a single *z*-plane were traced along the dendritic shafts where they are most focused, excluding dendritic spines. The mean GluA1 signal intensity was measured. GluA1 intensity values were normalized to SH condition.

### qRT-PCR

Mice were deeply anesthetized by isoflurane before rapid decapitation. Cortices were dissected into TRIzol reagent (Thermo Fisher Scientific), and RNA was extracted according to TRIzol's standard protocol. RNA was reverse transcribed to cDNA using the High-Capacity cDNA Reverse Transcription Kit (Thermo Fisher Scientific). qRT-PCR was performed with the CFX96 Touch Real-Time PCR Detection System (Bio-Rad) using PowerUp SYBR Green Master Mix (Thermo Fisher Scientific). Primers were designed using NCBI Primer-BLAST (Table 1). Samples were run in triplicate. Data were analyzed using the CFX Manager Software (Bio-Rad), using the ΔΔCt method. *Gapdh* was used as the reference gene itself, having no difference in expression between sample groups.

### Experimental design and statistical analysis

The design of each experiment is described. Statistical analysis and data visualization were performed using Prism (GraphPad Software). Outliers were identified using Prism's ROUT method, with *Q* = 0.5%. When indicated, data points were normalized to the appropriate control condition by dividing each value by the average of all control animals (water group, SH housing, or WT genotype). For *t* tests, variances were compared before choosing between Student's (if no significant difference) or Welch's *t* tests (if there is a significant difference). Pairwise *t* tests were used for measurements taken from the same animal. Additionally, one-way and two-way ANOVA with Tukey's or Sidak's multiple comparisons were used for pairwise comparisons. *p*-values are reported within graphs. In all graphs, data points represent individual animals unless otherwise stated in the figure legend.

Additional information on key materials is available in Table 1.

## Results

### Neuronal activity stimulates Sonic hedgehog signaling in vivo

**Figure 1.**
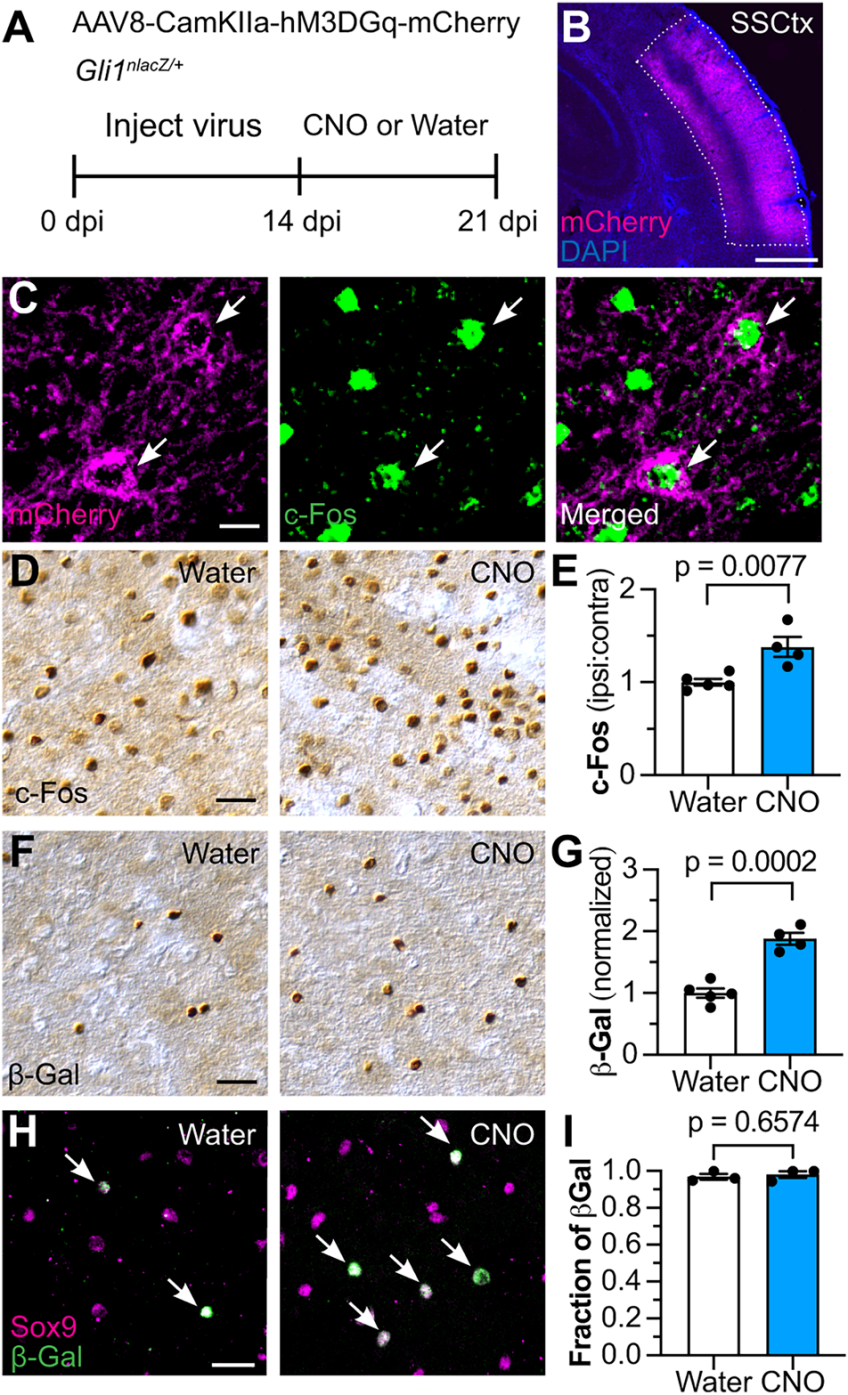
Neuronal activity stimulates Shh signaling. ***A***, Schematic depicting experimental approach. *Gli1^nlacZ/+^* mice were injected with AAV8-CamKIIα-hM3DGq-mCherry in the somatosensory cortex. Two weeks later, mice were fed CNO-medicated or unmedicated drinking water for 7 d before analysis. ***B***, The transfected region can be visualized with mCherry (magenta). Counterstained with DAPI (blue). SSCtx, somatosensory cortex. Scale bar, 250 μm. ***C***, mCherry (magenta, left panel) and c-Fos (green, middle panel) in the transfected region. Arrows point to cells coexpressing mCherry and c-Fos. Scale bar, 25 μm. ***D***, ***F***, Bright-field immunohistochemistry for c-Fos (***D***) and β-Gal (***F***) in the somatosensory cortex. Scale bar, 25 μm. ***E***, ***G***, Stereological quantification of c-Fos (***E***) and β-Gal (***G***) in the transfected region shows increased neural activity and increased Shh signaling in mice that received CNO compared with water-fed controls. *n* = 5 mice in water condition, *n* = 4 mice in CNO condition. Statistics: Student's *t* tests. ***H***, Immunofluorescent staining for Sox9 (magenta) and β-Gal (green) in the transfected region between water (left) and CNO (right) groups. Arrows point to β-Gal cells colabeled with Sox9. Scale bar, 25 μm. ***I***, Fraction of β-Gal-labeled cells colocalizing with Sox9 comparing between water and CNO groups. *n* = 3 mice in water condition, *n* = 3 mice in CNO condition. Statistics: Student's *t* test. In all graphs, data points represent individual animals; bars show mean ± SEM. Extended Data Figure 1-1 shows that chemogenetic stimulation does not increase the number of SHH-expressing neurons.

To examine whether neural activity stimulates Shh signaling in vivo, we used a chemogenetic approach to selectively excite cortical pyramidal neurons with the excitatory hM3DGq designer receptor exclusively activated by designer drugs (DREADD; Fig. 1*A*,*B*). We injected AAV8-CamKIIα-hM3DGq-mCherry virus (Alexander et al., 2009) into the somatosensory cortex of *Gli1^nlacZ/+^* mice which express nuclear lacZ at the *Gli1* locus (Bai et al., 2002). Because *Gli1* is a canonical target of Shh signaling, and because SHH is required for *Gli1* expression, transcriptional activation of *Gli1* acts as a reliable readout of Shh signaling (Bai et al., 2002; Hui and Angers, 2011). After 14 d, we treated mice with medicated drinking water containing the designer drug clozapine *N*-oxide (CNO) for 7 d to chronically activate the receptor (Armbruster et al., 2007). Transfected cells expressed c-*Fos* (Fig. 1*C*), and CNO-treated mice showed a significant increase in the number of c-Fos-labeled cells compared with virus-injected animals that were fed unmedicated drinking water, indicating successful activation of neurons (Fig. 1*D*,*E*). We observed a significant increase in the number of β-Gal+ cells in the somatosensory cortex in the CNO-treated group compared with untreated controls (Fig. 1*F*,*G*). Ninety-seven percent of β-Gal cells colocalized with Sox9 (Fig. 1*H*,*I*), a well-established marker for identifying astrocytes (Sun et al., 2017), consistent with our previous work identifying astrocytes as the predominant cell type expressing *Gli1* in the mature forebrain (Garcia et al., 2010; Gingrich et al., 2022). The fraction of cells identified as astrocytes does not change between water and CNO groups (Fig. 1*H*,*I*). Taken together, these data demonstrate that neuronal excitation stimulates Shh signaling in astrocytes in vivo.

10.1523/JNEUROSCI.1336-24.2025.f1-1 Figure 1-1 Chemogenetic stimulation of neuronal activity does not increase the number of *Shh*-expressing neurons. **(A)** Schematic depicting experimental approach to determine whether chemogenetic stimulation of neuronal activity increases the number of *Shh*-expressing cells. *Shh^CreER/+^*; Ai14 mice were injected with AAV5-hSyn-hM3DGq-HA targeting the somatosensory cortex. After 2 weeks, mice were fed CNO-medicated or unmedicated water for 10 days, receiving 3 doses of tamoxifen during the last 3 days. Tissues were analyzed seven days later. dpi, days post injection. **(B-D)** Immunolabeling for HA (green) shows the transduced region overlapping with tdTom labeled (magenta) cells in the cortex. Inset shown in (C). Individual cell shown at high power in (D). Counterstained with DAPI (blue). Scale bar, 25 μm. **(E)** The number of *Shh* neurons in the cortex between water control versus CNO. Data points represent individual animals, *n* *=* *2* mice in water group, *n* *=* *2* mice in CNO group. Bars show mean ± SEM. Statistic: Student’s t-test. Download Figure 1-1, TIF file.

In the cortex, *Shh* is expressed predominantly in a subset of excitatory pyramidal neurons in layer V (Harwell et al., 2012; Hill et al., 2019). To determine whether stimulation of neuronal activity increases the number or distribution of* Shh*-expressing cells, we also performed chemogenetic activation of cortical neurons in *Shh^CreER/+^*;Ai14 mice. We injected mice with AAV5-Syn-hM3DGq-HA virus and began CNO treatment 2 weeks later. Animals received CNO or unmedicated water for 10 d. To mark *Shh*-expressing cells, animals received tamoxifen over the last 3 d of CNO treatment and were analyzed 2 weeks later (Extended Data Fig. 1-1). There was no difference in the number or distribution of *Shh*-expressing cells between the CNO-treated and control animals (Extended Data Fig. 1-1), indicating an increase in the availability of SHH ligand, rather than an increase in the number of *Shh*-expressing neurons.

### Sensory experience stimulates Sonic hedgehog signaling

**Figure 2.**
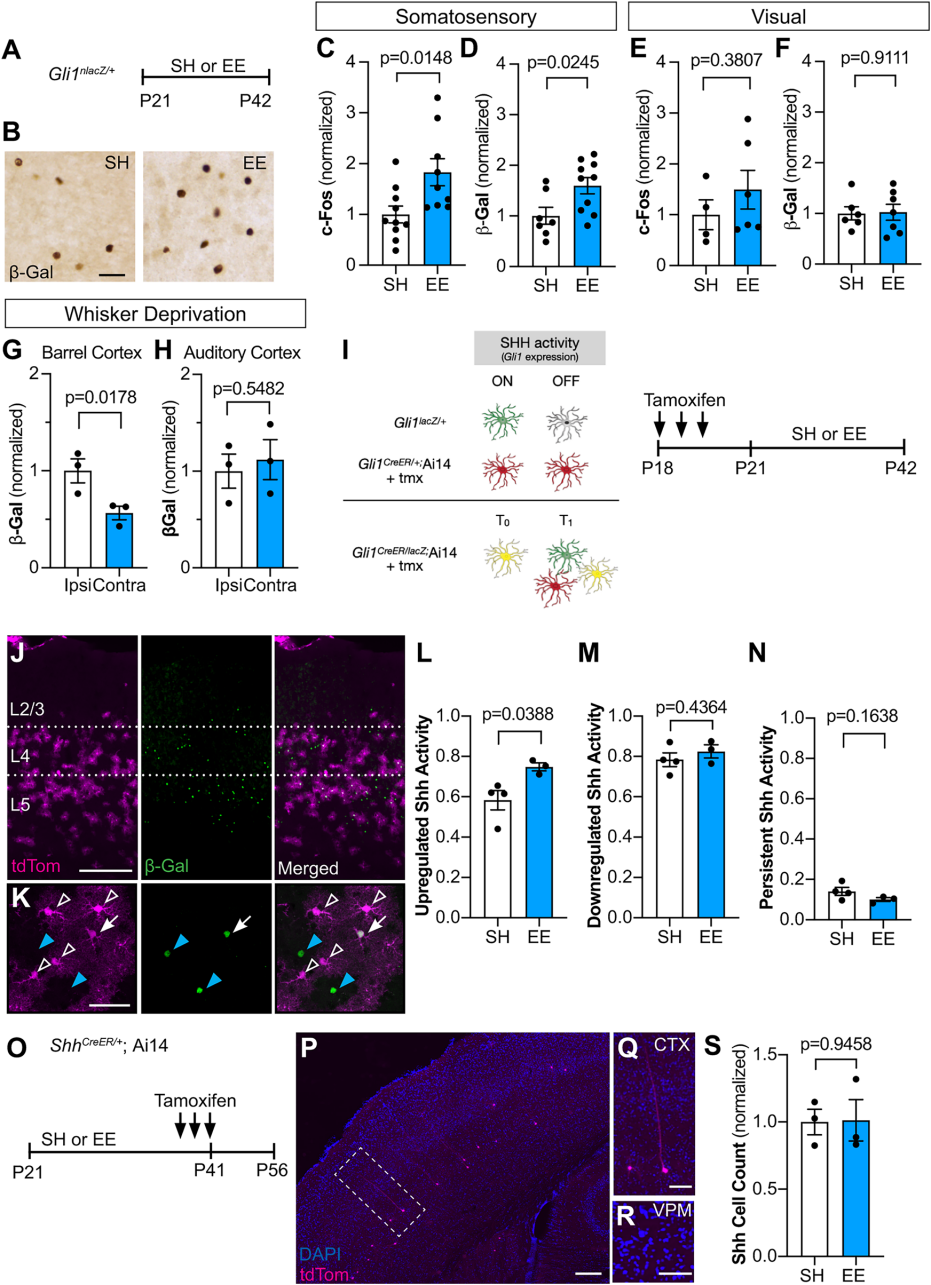
Enriched experience stimulates Shh signaling. ***A***, Experimental timeline. *Gli1^nlacZ/+^* mice were weaned into standard housing (SH) or enriched experience (EE) cages at P21 and analyzed at P42. ***B***, Representative bright-field immunohistochemistry for β-Gal in the somatosensory cortex. Scale bar, 50 μm. ***C***–***F***, Stereological quantification of cells expressing the immediate-early gene c-*Fos* (***C***, ***E***) or immunolabeled with β-Gal (***D***, ***F***) in the somatosensory (***C***, ***D***) or visual (***E***, ***F***) cortices. *n* = 4–10 mice in SH, *n* = 6–10 mice in EE. Statistics: Student's *t* tests. ***G***, ***H***, *Gli1^nlacZ/+^* mice were subjected to unilateral whisker trimming continuously from P21 and analyzed at P42. Stereological quantification of β-Gal cells from the ipsilateral (intact) and contralateral (deprived) barrel (***G***) and auditory (***H***) cortex. *n* = 3 mice. Statistics: paired *t* test. ***I***, Schematic depicting genetic strategy to label cells with temporally distinct Shh signaling (left panel) and timeline of experiment (right panel). In *Gli1^nlacZ/+^* mice, β-Gal expression labels cells with active Shh signaling. In *Gli1^CreER^*;Ai14 mice, Cre-mediated recombination promotes permanent expression of tdTom. In *Gli1^CreER/nlacZ^*;Ai14 mice, tdTom expression identifies cells with Shh signaling at baseline (*t*0) and β-Gal expression identifies Shh signaling in cells at the end of the experiment (*t*1). Double-labeled cells identify cells with persistent Shh signaling throughout the experiment. ***J***, ***K***, Low power (***J***) and high power (***K***) images showing tdTom expression (magenta, left panel) and immunostaining for β-Gal (green, middle panel; merged image, right panel) in the cortex of mice housed in EE as described in ***I***. Scale bar, 250 μm. ***K***, Individual cells show those with upregulation (blue arrowhead), downregulation (open arrowhead), and persistent (white arrow) Shh activity. Scale bar, 50 μm. ***L***–***N***, The fraction of β-Gal-labeled cells that are single labeled (upregulated Shh activity; ***L***), tdTom-labeled cells that are single labeled (downregulated Shh activity; ***M***), and all cells that are double labeled (persistent Shh activity; ***N***) in SH or EE. *n* = 4 mice in SH, *n* = 3 mice in EE. ***O***, Schematic depicting experimental approach: P21 *Shh^CreER/+^*;Ai14 mice were weaned into SH or EE cages and received three doses of tamoxifen from P39 to P41. Tissues were analyzed 2 weeks after the last tamoxifen dose. ***P***–***R***, Low power image showing tdTom-labeled cells (magenta) in *Shh^CreER/+^*;Ai14 mice. Labeled cells are found predominantly in layer V. Inset shows individual cells with neuronal morphology in the cortex (***Q***). Note the absence of labeled cells in the VPM (***R***). Counterstained with DAPI (blue). Scale bars: ***P***, 50 μm; ***Q***, ***R***, 20 μm. ***S***, The number of *Shh* neurons in the cortex in SH and EE mice. *n* = 3 mice in SH, *n* = 3 mice in EE. In all figures, data points represent individual animals; bars show mean ± SEM.

We next examined whether a more physiological stimulus can increase Shh signaling. To test this, we placed *Gli1^nlacZ/+^* mice in an enriched sensory environment designed to stimulate active whisking (Yang et al., 2009). Mice were weaned into large rat cages from which strings of beads were hung from the wire cage lid in a manner requiring constant interaction at Postnatal day 21 (P21). Control mice were weaned into similar rat cages with only standard nesting materials and no beads. Mice were housed in the enriched environment (EE) or standard housing (SH) conditions for 3 weeks (Fig. 2*A*). A significant increase in the number of cells labeled with c-Fos was observed in the somatosensory cortex of mice housed in EE compared with SH conditions (Fig. 2*C*), confirming increased activity in this region. Mice housed in EE showed a significant increase in the number of β-Gal-labeled cells compared with control mice housed in SH conditions (Fig. 2*B*,*D*), indicating an increase in Shh activity in response to sensory experience. Notably, there was no difference in either c-Fos or β-Gal labeling between EE and SH conditions in the visual cortex (Fig. 2*E*,*F*), demonstrating that sensory stimulation does not produce a generalized increase in Shh signaling but rather selectively increases activity of the pathway in circuits that are stimulated by experience. Because sensory activity stimulates Shh signaling, we next asked whether sensory deprivation lowers the activity of the pathway. To do this, we performed unilateral whisker trimming on P21 *Gli1^nlacZ/+^* mice for 3 weeks, starting at birth. We analyzed the deprived, contralateral hemisphere and compared it to the intact, ipsilateral hemisphere. We observed significantly fewer β-Gal+ cells in the contralateral, deprived hemisphere compared with the ipsilateral, intact hemisphere (Fig. 2*G*). Analysis of the auditory cortex showed whisker trimming had no effect on Shh signaling in this region (Fig. 2*H*). Taken together, these data demonstrate that activation of distinct sensory circuits selectively stimulates Shh signaling in cortical astrocytes in a circuit-specific manner.

To precisely identify cells that show increased Shh activity following experience, we combined the direct reporter approach with a tamoxifen-dependent labeling strategy, enabling us to monitor temporally distinct populations of cells experiencing Shh signaling. We crossed *Gli1^nlacZ/+^* mice with *Gli1^CreER/+^*;Ai14 mice carrying the Cre-dependent Ai14 reporter allele expressing tdTomato (tdTom; *Gli1^nlacZ/CreER^*;Ai14), enabling identification of cells with Shh signaling at the start and conclusion of sensory enrichment. Because *lacZ* expression is directly regulated by the transcriptional activity of *Gli1*, cells actively transducing SHH can be monitored by immunohistochemistry for β-Gal, the protein product of the *lacZ* gene. In contrast, tamoxifen-dependent, Cre-mediated recombination produces permanent expression of tdTom in cells, even after Shh activity is downregulated (Fig. 2*I*). Thus, in *Gli1^nlacZ/CreER^*;Ai14 mice, *lacZ* expression reflects active Shh signaling whereas tamoxifen-dependent Cre-mediated expression of tdTom reflects historical signaling. Although these mice are effectively *Gli1* nulls, GLI1 is dispensable for Shh signaling due to the redundant activator function of GLI2 (Bai et al., 2002; Niewiadomski et al., 2019), and we did not observe any gross anatomical or behavioral phenotypes, consistent with previous studies (Bai et al., 2002; Gingrich et al., 2022). To mark cells with Shh signaling at baseline, mice received tamoxifen before placing them in EE or SH housing for 3 weeks (Fig. 2*I*). We first examined the population of β-Gal+ cells and found a large fraction of cells that were negative for tdTom (Fig. 2*J*,*K*), indicating Shh activity in a population of cells distinct from those marked at the start of the experiment. Notably, there was a significant increase in single-labeled β-Gal cells after EE compared with SH (Fig. 2*L*). Whereas 59% of β-Gal-labeled cells were single labeled in SH mice, this fraction increased to 76% in EE mice (Fig. 2*L*) demonstrating experience-dependent activation of the pathway in cells distinct from those at baseline. These cells were found in layers IV and V (Fig. 2*J*), suggesting that experience-dependent Shh activity remains localized to deep cortical layers, consistent with the localization of *Shh*-expressing neurons in layer V (Garcia et al., 2010; Harwell et al., 2012). We next examined the population of cells expressing tdTom. tdTom+ cells were localized primarily in layers IV and V and showed a bushy morphology characteristic of protoplasmic astrocytes, consistent with previous studies (Garcia et al., 2010; Hill et al., 2019; Fig. 2*J*,*K*). In SH mice, a large proportion of tdTom+ cells did not colabel with β-Gal (Fig. 2*K*,*M*), indicating downregulation of pathway activity. This fraction did not change after exposure to EE (Fig. 2*M*). We also observed a third population of cells, those coexpressing both tdTom and β-Gal, suggesting either persistent or recurrent Shh activity in these cells (Fig. 2*K*,*N*). This fraction was similar between SH and EE conditions (Fig. 2*N*). These data suggest that Shh activity in individual cells is highly dynamic and that over a 3-week period, individual cells are not likely to experience persistent Shh signaling.

To examine whether enriched experience stimulates *Shh* expression, we housed *Shh^CreER/+^*;Ai14 mice in EE or SH conditions. Mice received tamoxifen over the last 3 d of the housing experience and were analyzed 2 weeks later (Fig. 2*O*). Similar to our chemogenetic approach, labeled neurons were found primarily in layer V (Fig. 2*P*,*Q*), and we found no difference in the number of *Shh*-expressing neurons between SH and EE (Fig. 2*S*). These data demonstrate that while transduction of Shh signaling in astrocytes is dynamically modulated between individual cells, the SHH ligand is derived from a defined population of neurons. The increase in Shh signaling may arise from an increase in SHH release or from increased diffusion in the extracellular space. Because astrocytes in layer IV show high levels of Shh activity, we also examined the ventral posterior medial nucleus of the thalamus (VPM) where thalamocortical neurons projecting to layer IV of the barrel cortex reside (Staiger and Petersen, 2020). We detected few, if any, labeled neurons in this region (Fig. 2*R*) suggesting that Shh signaling in cortical astrocytes is initiated by ligand released from local neurons.

**Figure 3.**
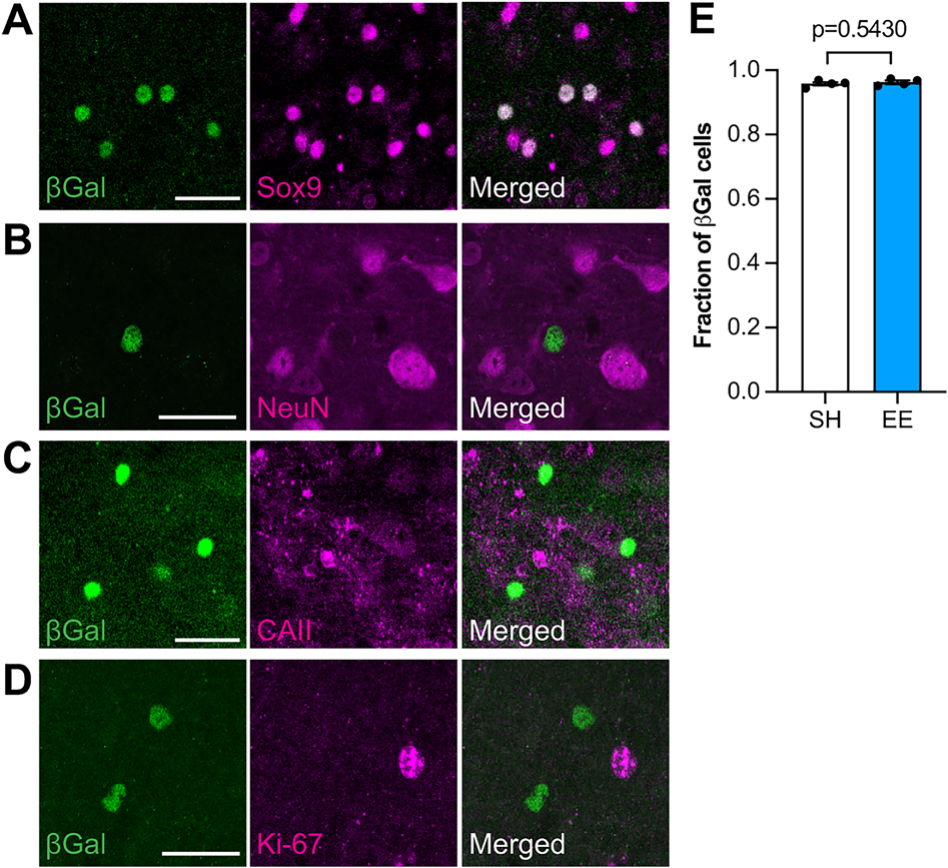
Enriched experience stimulates Shh activity in astrocytes. ***A***–***D***, Immunofluorescence for β-Gal (green) and Sox9 (***A***), NeuN (***B***), CAII (***C***), or Ki-67 (***D***; magenta) in the cortex of *Gli1^nlacZ/+^* mice. Counterstained with DAPI (blue). Merged images shown in the right panels. Scale bar, 25 μm. ***E***, The fraction of β-Gal-labeled cells colocalizing with Sox9 does not change between SH and EE. Data points represent individual animals, *n* = 4 mice in SH, *n* = 4 mice in EE, 100–400 β-Gal cells analyzed per animal. Bars show mean ± SEM. Statistic: Student's *t* test.

We examined the identity of β-Gal-labeled cells with cell-type–specific markers. Colocalization with Sox9, CAII, and NeuN to identify astrocytes, oligodendrocytes, and neurons, respectively, showed that 96% of β-Gal-labeled cells correspond to astrocytes in both SH and EE (Fig. 3*A*–*C*,*E*) consistent with our previous observation that astrocytes are the primary targets of canonical Shh signaling in the adult brain (Garcia et al., 2010). Β-Gal+ cells did not express Ki-67 (Fig. 3*D*), ruling out the possibility that the observed increase in cell number is due to proliferation.

### Sensory experience dynamically modulates Sonic hedgehog signaling

**Figure 4.**
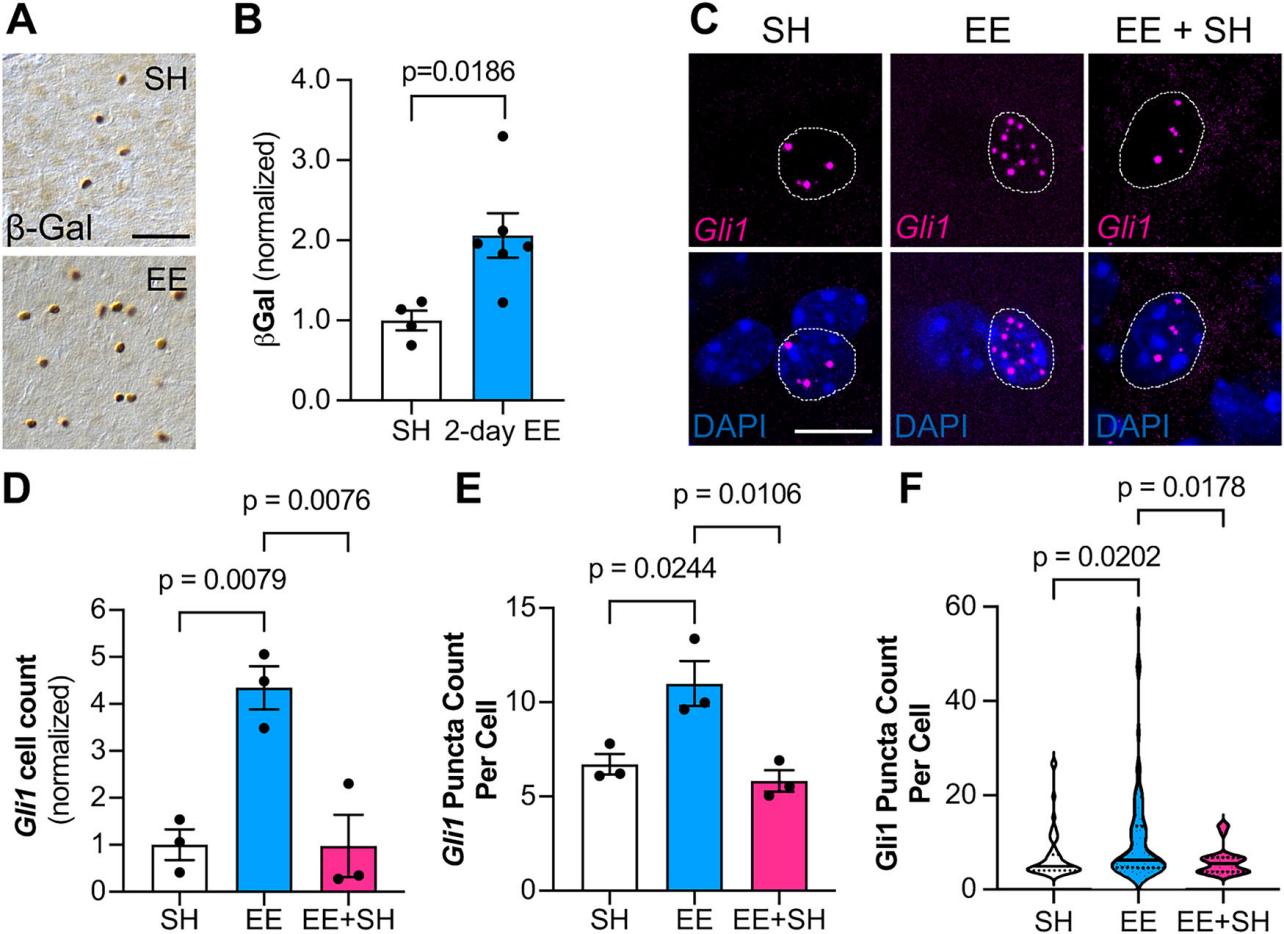
Shh signaling responds rapidly to sensory activation and is not persistent. ***A***, Bright-field immunohistochemistry for β-Gal in the somatosensory cortex of *Gli1^nlacZ/+^* mice housed in SH (top panel) or EE (bottom panel) for 2 d. Scale bar, 50 μm. ***B***, Stereological quantification of β-Gal shows increased Shh activity in the somatosensory cortex after 2 d of EE. Bars represent mean ± SEM. Data points represent individual animals, *n* = 4 mice in SH, *n* = 6 mice in EE. Statistics: Student's *t* test. ***C***, Representative images of fluorescent in situ hybridization for *Gli1* mRNA in the deep layers of the cortex from SH, EE, and EE and SH housing conditions. Dotted line outlines individual DAPI nuclei with *Gli1* mRNA. Scale bar, 10 μm. ***D***, *Gli1+* cell count in layers IV and V of the cortex from mice housed in SH or EE for 4 d or EE for 2 d followed by SH for 2 d (EE + SH). In each condition, 1,000–2,000 DAPI cells were analyzed. Bars represent mean ± SEM. Data points represent individual animals, *n* = 3 mice per condition. Statistics: one-way ANOVA with Tukey's multiple comparisons. ***E***, ***F***, Quantification of *Gli1* puncta in individual cells in layers IV and V from SH, EE, and EE and SH conditions. Bar graphs (***E***) show data by animal, and violin plots (***F***) show data by individual cells. In each condition, 400–600 cells were analyzed, and only cells with >3 puncta were included in quantification; *n* = 3 mice per condition. Bars (***E***) represent mean ± SEM, and violin plots (***F***) show median ± interquartile ranges. Statistics: one-way ANOVA with Tukey's multiple comparisons.

We next examined whether the experience-induced increase in Shh activity reflects a long-term change in Shh signaling. We first determined whether a much shorter time frame was sufficient to increase Shh activity. We housed *Gli1^nlacZ/+^* mice in either SH or EE for 2 d starting at P21. Mice housed in EE showed a significant increase in β-Gal+ cells compared with SH controls (Fig. 4*A*,*B*), suggesting that brief exposure to sensory enrichment is sufficient to stimulate Shh signaling. To examine whether experience-dependent Shh signaling persists after removal of the stimulus, we housed *Gli1^nlacZ/+^* mice in EE for 2 d, then housed them in SH condition for 2 d, and compared *Gli1* activity with mice continuously housed in EE or SH conditions for 4 d. To rule out the possibility that any observed differences in β-Gal number may reflect perdurance of the protein rather than sustained Shh activity, we used single-molecule fluorescent in situ hybridization (smFISH) by RNAscope to more directly measure *Gli1* transcripts (Fig. 4*C*). In mice continuously housed in EE, we found an increase in cells expressing *Gli1* compared with SH control mice (Fig. 4*C*,*D*). However, in mice returned to SH after 2 d of EE, *Gli1* expression returned to baseline (Fig. 4*C*,*D*). Notably, individual cells expressing *Gli1* also showed a strong increase in the number of transcripts in the nucleus after exposure to EE compared with SH-housed mice (Fig. 4*C*,*E*,*F*). This suggests that neural activity not only stimulates Shh signaling in new cells but also further increases the activity of the pathway in cells with baseline Shh signaling. These data demonstrate that Shh signaling is highly dynamic and responsive to changing experiences.

**Figure 5.**
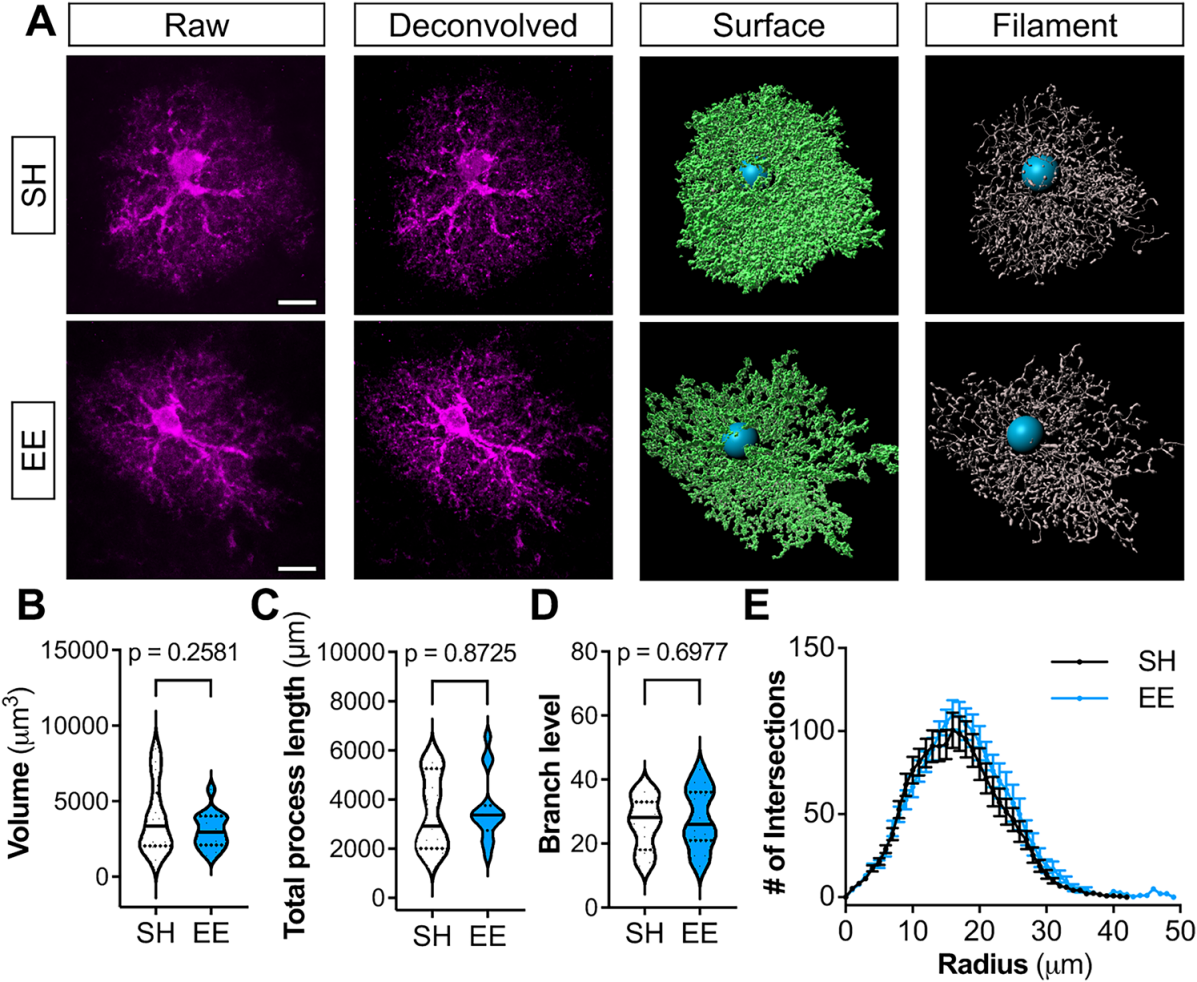
Enriched experience does not alter *Gli1* astrocyte morphology. ***A***, Representative images for *Gli1* astrocytes for mice housed in SH (top) or EE (bottom) in the somatosensory cortex. ***B***–***D***, Morphological quantification of volume (***B***), process length (***C***), and branch level (***D***). Violin plots show data by individual astrocytes; median ± interquartile, *n* = 3 mice in SH, *n* = 3 mice in EE, five astrocytes analyzed per animal. Statistics: Student's *t* tests. ***E***, Sholl analysis of astrocytes from SH versus EE conditions. Data represent mean ± SEM by animal; *n* = 3 animals, five astrocytes analyzed per animal.

To determine whether experience-dependent Shh signaling promotes morphological plasticity of astrocytes, we analyzed the morphologies of tdTom-labeled astrocytes in the barrel cortex of P21 *Gli1^CreER^*;Ai14 mice after SH or EE. We reconstructed astrocytes in 3D using Imaris and found no differences in total volume, branch level, process length, or morphological complexity, as measured by Sholl analysis (Fig. 5) between SH and EE conditions.

### Astrocytic Sonic hedgehog signaling is required for deep-layer synaptic plasticity

**Figure 6.**
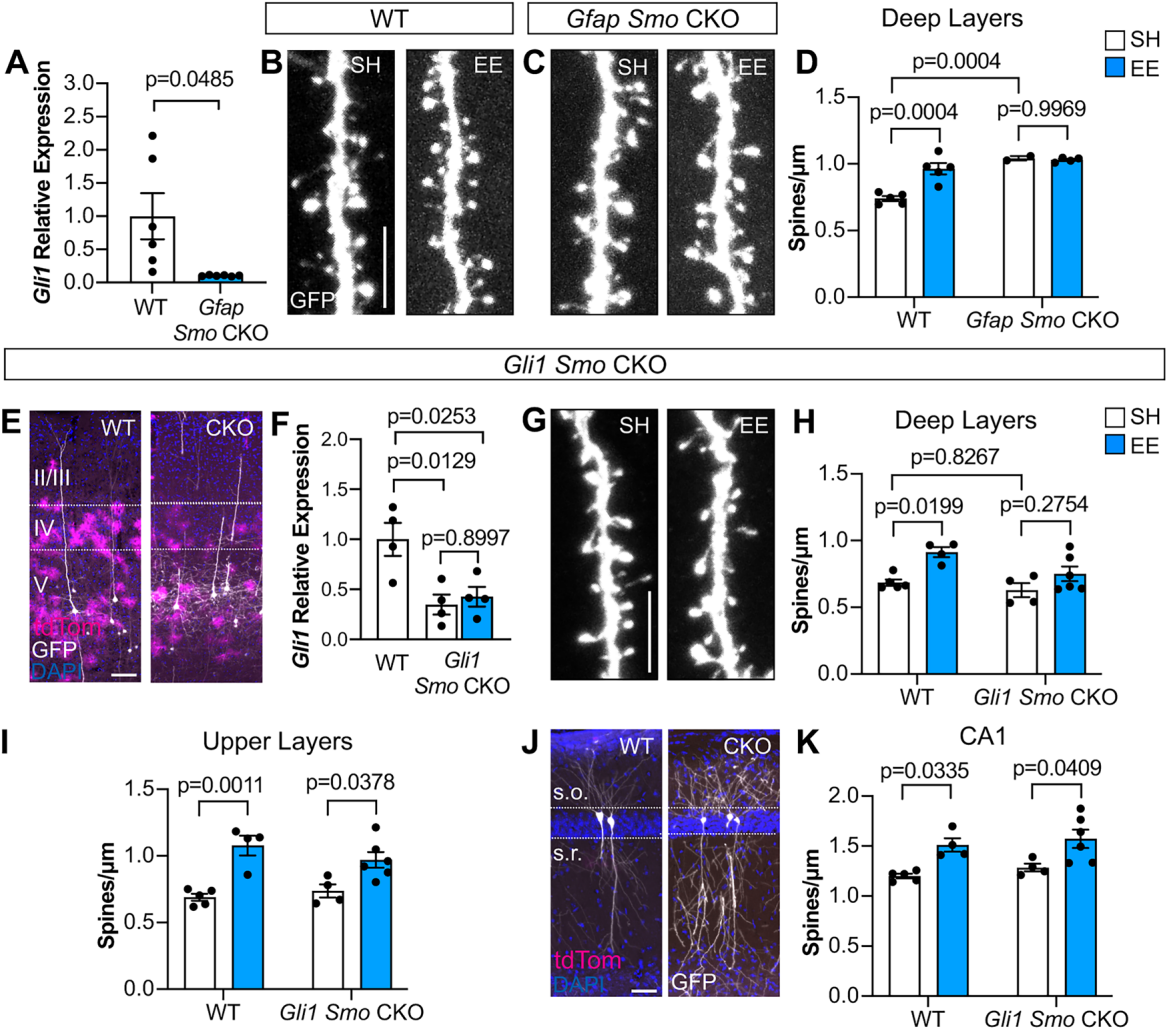
Experience-dependent structural plasticity requires Shh signaling. ***A***, qRT-PCR for *Gli1* expression in WT and *Gfap Smo* CKO mice. *n* = 6 mice per condition. Statistics: Welch's *t* test. ***B***, ***C***, Representative dendritic segments in deep layers of WT (***B***) and *Gfap Smo* CKO (***C***) mice housed in SH or EE. Scale bar, 5 μm. ***D***, Spine density of dendritic segments in deep layers of WT and *Gfap Smo* CKO mice housed in SH or EE. *n* = 5 WT mice in SH, *n* = 5 WT mice in EE, *n* = 2 *Gfap Smo* CKO mice in SH, *n* = 4 *Gfap Smo* CKO mice in EE, 7–12 dendritic segments analyzed per animal. Statistics: two-way ANOVA with Tukey's multiple comparisons. ***E***, tdTom (magenta)-labeled astrocytes and GFP (white)-labeled neurons, counterstained with DAPI (blue), in the cortex of WT (left) and *Gli1 Smo* CKO (right) mice. Scale bar, 100 μm. ***F***, qRT-PCR for *Gli1* expression in WT and *Gli1 Smo* CKO mice housed in SH or EE. *n* = 4 mice per condition. Statistics: one-way ANOVA with Tukey's multiple comparisons. ***G***, Dendritic segments from *Gli1 Smo* CKO mice housed in SH or EE. Scale bar, 5 μm. ***H***, ***I***, Spine density of dendritic segments in deep layers (***H***) or upper layers (***I***) from WT and *Gli1 Smo* CKO mice housed in SH or EE. WT data pooled from various littermate control animals with and without Cre or tamoxifen (Extended Data Fig. 6-1). *n* = 5 WT mice in SH, *n* = 4 mice in EE, *n* = 4 *Gli1 Smo* CKO mice in SH, *n* = 6 *Gli1 Smo* CKO mice in EE, 3–10 dendritic segments analyzed per animal. Statistics: one-way ANOVA with Tukey's multiple comparisons. ***J***, tdTom (magenta)-labeled and GFP (white)-labeled cells in area CA1 of the hippocampus from WT and *Gli1 Smo* CKO mice, counterstained with DAPI (blue). Area CA1 contains few tdTom-labeled cells reflecting the absence of Shh activity in hippocampal astrocytes. s.o., stratum oriens; s.r., stratum radiatum. Scale bar, 50 μm. ***K***, Spine density from apical dendrites of CA1 neurons in WT and *Gli1 Smo* CKO mice housed in SH or EE. *n* = 5 WT mice in SH, *n* = 4 mice in EE, *n* = 4 *Gli1 Smo* CKO mice in SH, *n* = 6 *Gli1 Smo* CKO mice in EE, three dendritic segments analyzed per animal. Statistics: one-way ANOVA with Tukey's multiple comparisons. In all graphs, data points represent individual animals; bars show mean ± SEM.

Sensory experience promotes structural plasticity of cortical synapses (Yang et al., 2009). To determine whether experience-dependent Shh signaling in astrocytes promotes synaptic plasticity, we examined dendritic spines of layer V cortical neurons. Excitatory synapses are localized predominantly on dendritic spines (Nimchinsky et al., 2002), which demonstrate remarkable plasticity in response to sensory experience (Zuo et al., 2005; Yang et al., 2009; Landers et al., 2011; Jung and Herms, 2014; Chen et al., 2015). To determine whether Shh signaling plays a role in astrocyte modulation of experience-dependent plasticity, we examined the spine density of cortical neurons in conditional knock-out (CKO) mice lacking *Smo*, the obligatory coreceptor for transduction of Shh signaling, selectively in astrocytes. We used *Gfap-Cre*;*Smo^fl/fl^* mice (*Gfap Smo* CKO) in which *Smo* is deleted in *Gfap*-expressing cells, effectively abolishing Shh signaling in all astrocytes beginning at birth (Garcia et al., 2010; Hill et al., 2019). These mice also carry a *Thy1*-GFPm allele which labels a subset of layer V pyramidal neurons (Feng et al., 2000), enabling the visualization of individual dendrites and spines. *Gli1* expression measured by qRT-PCR showed a 90% reduction in *Gfap Smo* CKO mice compared with WT littermate controls lacking Cre, confirming effective ablation of Shh activity (Fig. 6*A*). We analyzed apical dendrites of layer V pyramidal neurons in the barrel cortex, focusing on dendritic segments in layers IV and V where high levels of Shh activity are found. WT (Cre-negative littermates) mice housed in EE conditions showed a significant increase in the density of protrusions compared with control SH mice (Fig. 6*B*,*D*), demonstrating that enriched sensory experience promotes structural synaptic plasticity, consistent with previous studies (Yang et al., 2009; Landers et al., 2011). We previously demonstrated that *Gfap Smo* CKO mice showed an elevated spine density arising from deficits in the developmental elimination of synapses (Hill et al., 2019). Here, we find that sensory enrichment did not produce any further increases in spine density (Fig. 6*C*,*D*), consistent with a requirement for astrocytic Shh signaling in structural plasticity. Alternatively, this may instead reflect a limit beyond which no further spines can be added. Shh signaling acts on astrocyte progenitor cells in the perinatal subventricular zone that generate cortical astrocytes (Gingrich et al., 2022). Because recombination begins at P0 in cortical astrocyte progenitors in this *Gfap-Cre* line (Garcia et al., 2010), this impaired plasticity may reflect developmental perturbation of cortical astrocyte development.

10.1523/JNEUROSCI.1336-24.2025.f6-1 Figure 6-1 Wild-type littermate controls for comparison with *Gli1 Smo* CKO were pooled from different conditions. **(A)** Spine density of deep layer dendritic segments in wild-type littermate controls of *Gli1 Smo* CKO animals housed in SH, including Cre- mice with and without tamoxifen and Cre + mice without tamoxifen. Animals were subsequently pooled as WT for comparison with *Gli1 Smo* CKO. **(B)** Spine density of deep layer dendritic segments in wild-type littermate controls of *Gli1 Smo* CKO animals housed in EE, including Cre- and Cre + without tamoxifen. Animals were subsequently pooled as WT for comparison with *Gli1 Smo* CKO. Data points represent individual animals; bars show mean ± SEM. Download Figure 6-1, TIF file.

To more directly test whether Shh signaling in mature astrocytes is required for synaptic plasticity, we generated *Gli1^CreER/+^*;*Smo^fl/fl^*;Ai14; *Thy1*-GFPm mice (*Gli1 Smo* CKO), in which tamoxifen-dependent *Smo* deletion enables temporal control. Tamoxifen administration at P14 or older limits *Smo* deletion to postmitotic, *Gli1*-expressing cells primarily in layers IV and V (Fig. 6*E*; Gingrich et al., 2022). *Gli1 Smo* CKO mice received tamoxifen over 3 d between P18 and P20 and were housed in EE or SH at P21 for 2 d. We measured *Gli1* expression of cortical tissues by qRT-PCR and detected a 66% reduction in *Gli1* transcripts in *Gli1 Smo* CKO mice compared with WT littermate controls that were Cre-negative, confirming effective interruption of Shh signaling (Fig. 6*F*). Cre-negative littermates with and without tamoxifen, as well as Cre-positive littermates without tamoxifen, showed no significant difference in dendritic spine density and were subsequently pooled as WT controls (Extended Data Fig. 6-1). WT mice housed in EE showed a significant increase in spine density compared with SH controls (Fig. 6*H*). In contrast, *Gli1 Smo* CKO mice failed to show a significant difference in spine density between housing conditions, demonstrating a requirement for astrocytic Shh signaling in structural plasticity (Fig. 6*G*,*H*). To determine whether plasticity is perturbed globally, we analyzed regions where Shh activity in astrocytes is low or absent. Notably, analysis of dendritic segments in the upper layers (layers II/III) of both WT and *Gli1 Smo* CKO mice showed a significantly higher spine density in EE compared with SH (Fig. 6*I*), suggesting that structural plasticity of these synapses is intact. We also analyzed apical dendrites of pyramidal neurons in area CA1 of the hippocampus, which lack astrocytic Shh activity (Hill et al., 2019; Fig. 6*J*). *Gli1 Smo* CKO mice housed in EE also showed a significant increase in spine density compared with SH (Fig. 6*K*), further supporting the specificity of this mouse model. Taken together, these data demonstrate that Shh signaling in astrocytes promotes structural plasticity induced by enriched sensory experience. It should be noted that in these mice, low levels of Shh stimulation that are insufficient to promote transcriptional activation of *Gli1* would leave *Smo* intact in the upper-layer and hippocampal astrocytes. Nevertheless, our observation that plasticity is impaired specifically in synapses where astrocytic Shh activity is high suggests that SHH-dependent astrocyte modulation of synaptic plasticity is mediated by local interactions.

**Figure 7.**
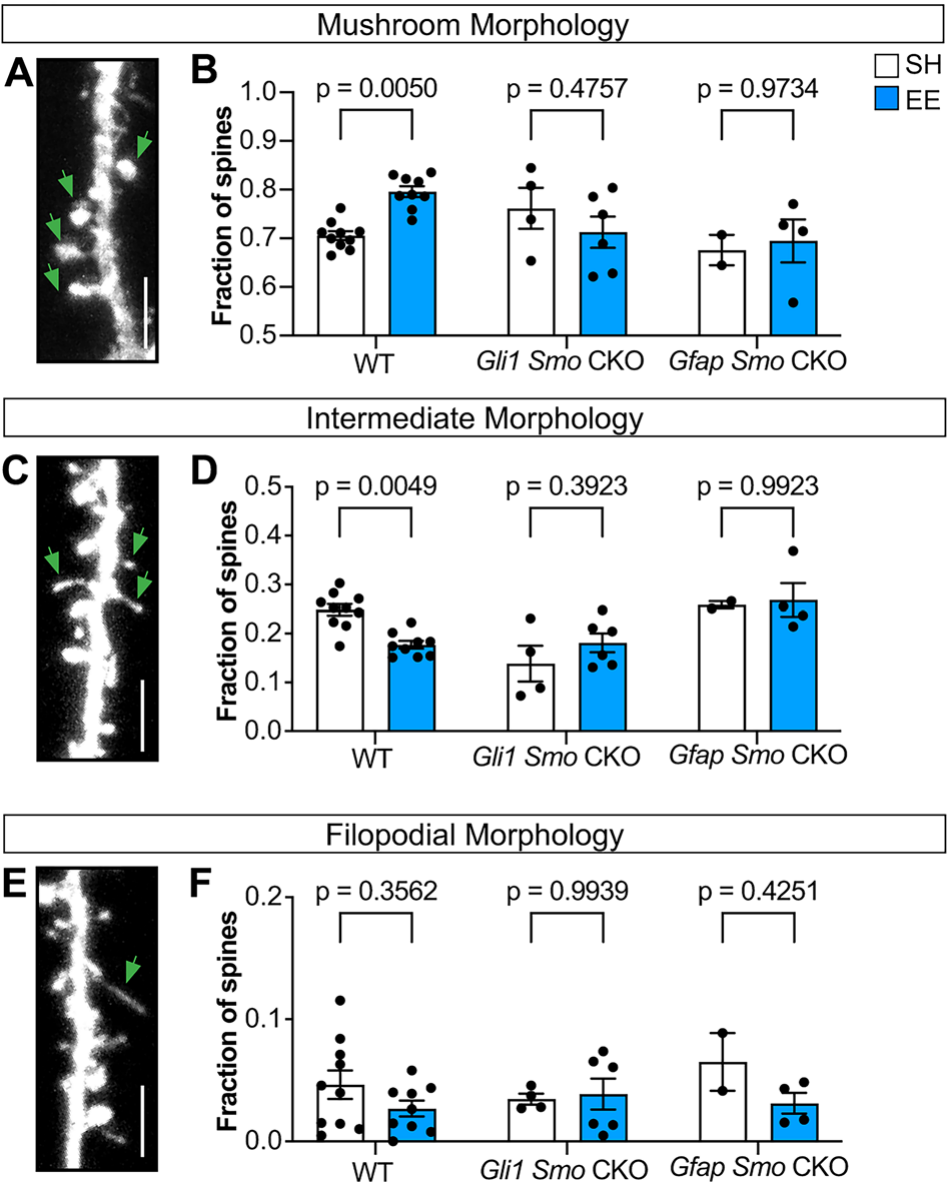
Experience-dependent spine enlargement requires Shh activity. ***A***, ***C***, ***E***, Representative examples of dendritic protrusions categorized as mushroom spines (***A***), intermediate spines (***C***), and filopodia (***D***). Scale bar, 5 μm. ***B***, ***D***, ***F***, Fraction of mushroom spines (***B***), intermediate spines (***D***), and filopodia (***F***) from WT, *Gli1 Smo* CKO, and *Gfap Smo* CKO mice housed in SH versus EE. At least 300 spines from three dendritic segments were analyzed per animal. *n* = 9–10 WT animals, *n* = 4–6 *Gli1 Smo* CKO animals, *n* = 2–4 *Gfap Smo* CKO animals per housing condition. Data points represent individual animals; bars show mean ± SEM. Statistics: two-way ANOVA with Sidak's multiple comparisons.

We next analyzed spine morphology, classifying them into mushroom, thin, and filopodial morphologies, and examined whether the proportion of spines in each class changed in response to experience and Shh signaling. Spine morphology is associated with synapse size and strength (Arellano et al., 2007; Holtmaat and Svoboda, 2009). In WT mice housed in EE, we observed an increase in the proportion of spines with a mushroom morphology (Fig. 7*A*,*B*), consistent with previous reports demonstrating that activity promotes the enlargement of spines (Matsuzaki et al., 2004). This was accompanied by a concomitant reduction in the fraction of intermediate spines, while the fraction of filopodia was unchanged (Fig. 7*C*–*F*). In contrast, the proportion of mushroom spines was similar between EE and SH conditions in both *Gfap Smo* CKO and *Gli1 Smo* CKO mice (Fig. 7*B*) indicating that selective perturbation of Shh signaling in astrocytes interferes with activity-dependent spine enlargement. These mice also showed no difference in the fraction of spines with intermediate or filopodial morphology between EE and SH conditions (Fig. 7*D*,*F*).

### Astrocytic Sonic hedgehog signaling is required for activity-dependent AMPA receptor synaptic composition

**Figure 8.**
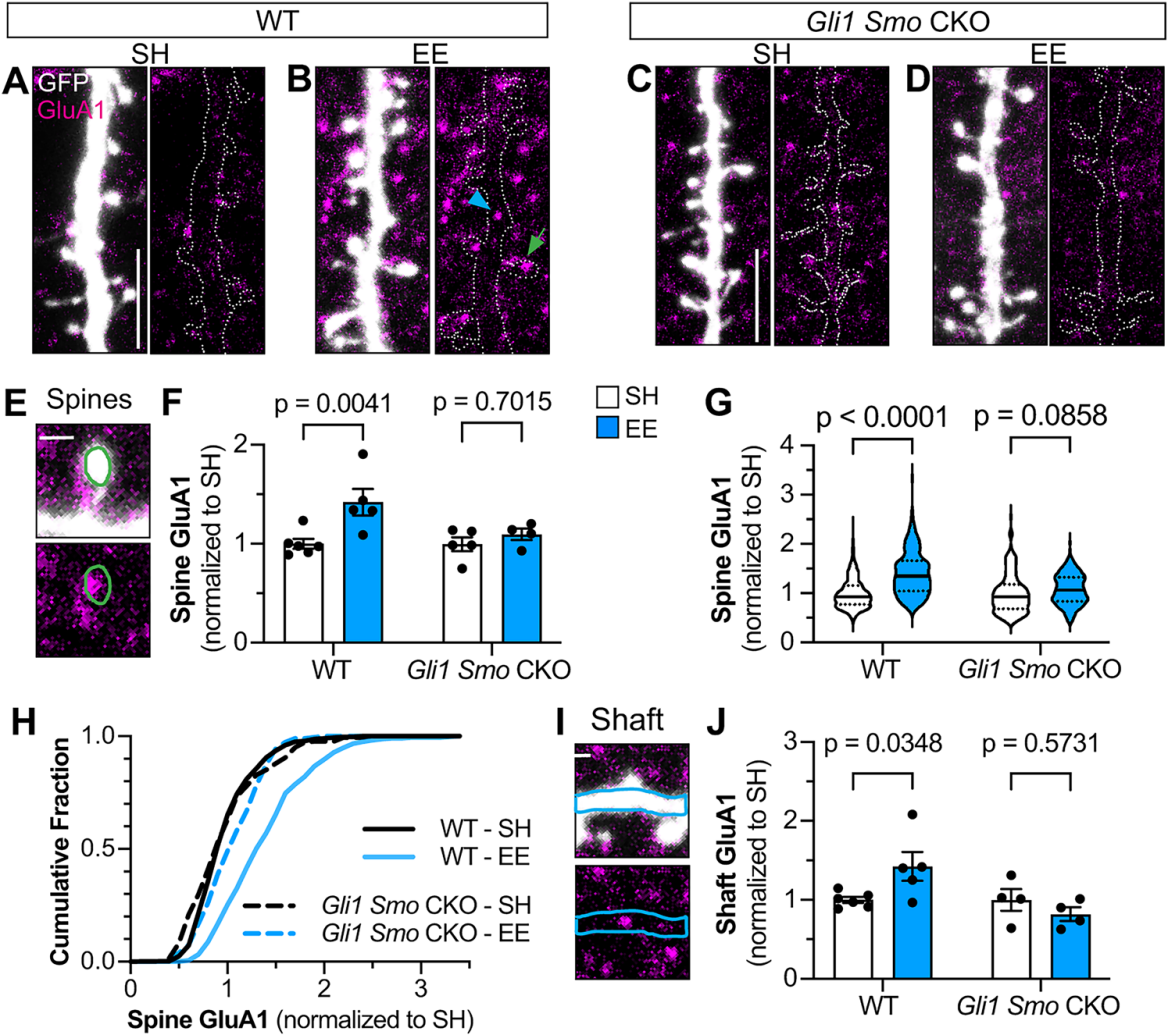
Experience-dependent increase of GluA1-AMPAR in spines requires Shh signaling. ***A***–***D***, Representative images of GluA1 immunostaining in layer IV and V apical dendrites from WT (***A***, ***B***) and *Gli1 Smo* CKO (***C***, ***D***) housed in SH (left) versus EE (right). The green arrow identifies the individual dendritic spine shown in ***E***. The blue arrowhead identifies the dendritic shaft segment shown in ***I***. Scale bar, 5 μm. ***E***, Example of ROI drawn at dendritic spine (green trace) to measure GluA1 intensity. Scale bar, 1 μm. ***F***, ***G***, GluA1 intensity at dendritic spines in WT and *Gli1 Smo* CKO mice housed in SH versus EE. Data points represent individual animals, *n* = 6 WT mice in SH, *n* = 5 WT mice in EE, *n* = 5 *Gli1 Smo* CKO mice in SH, *n* = 4 *Gli1 Smo* CKO mice in EE (***F***). Bars show mean ± SEM. Statistics: two-way ANOVA with Sidak's multiple comparisons. Data from all spines shown in (***G***), *n* = 329 spines in WT-SH, *n* = 340 spines in WT-EE, *n* = 181 spines in *Gli1 Smo* CKO-SH, *n* = 230 spines in *Gli1 Smo* CKO-EE. Solid and dashed lines show median ± interquartile ranges, respectively. Statistics: two-way ANOVA with Sidak's multiple comparisons. ***H***, Cumulative frequency distribution of GluA1 in spines plotted in ***F*** and ***G***. ***I***, Example of ROI drawn at dendritic shaft (blue trace) to measure GluA1 intensity. Scale bar, 1 μm. ***J***, Comparison of GluA1 intensity in dendritic shaft in WT and *Gli1 Smo* CKO mice housed in SH versus EE. Data points represent individual animals, *n* = 6 WT mice in SH, *n* = 5 WT mice in EE, *n* = 4 *Gli1 Smo* CKO mice in SH, *n* = 4 *Gli1 Smo* CKO mice in EE. Bars show mean ± SEM. Statistics: two-way ANOVA with Sidak's multiple comparisons.

Both neuronal activity and astrocytes regulate the trafficking of α-amino-3-hydroxy-5-methyl-4-isoxazole propionic acid receptors (AMPARs) at the synapse (Takahashi et al., 2003; Makino and Malinow, 2011; Allen et al., 2012; Zhang et al., 2015; Blanco-Suarez et al., 2018; Tan et al., 2020). Astrocytes secrete glypicans 4 and 6 which increase the surface expression of GluA1-containing AMPARs (Allen et al., 2012). To examine whether astrocytic Shh signaling regulates experience-dependent AMPAR composition at the synapse, we examined GluA1 expression in GFP-labeled spines following exposure to EE (Fig. 8*A*–*E*; Graves et al., 2021). In WT mice, there was a significant increase in GluA1 at dendritic spines in mice housed in EE compared with SH (Fig. 8*F*–*H*), supporting an experience-dependent increase, consistent with previous reports (Graves et al., 2021). In contrast, this experience-dependent increase of GluA1 was not observed in *Gli1 Smo* CKO mice (Fig. 8*F*–*H*), demonstrating a requirement for astrocytic Shh signaling in the experience-dependent increase of GluA1 at synapses. We also measured GluA1 intensity in dendritic shafts, which also increases following sensory stimulation (Zhang et al., 2015). Similar to dendritic spines, WT mice housed in EE showed an increase in GluA1 within the dendritic shaft which was not observed in *Gli1 Smo* CKO mice (Fig. 8*I*,*J*). Taken together, these data suggest that Shh activity in deep-layer astrocytes promotes activity-dependent recruitment of GluA1 to synapses.

### Sonic hedgehog signaling upregulates expression of astrocyte-derived synapse-modifying cues

**Figure 9.**
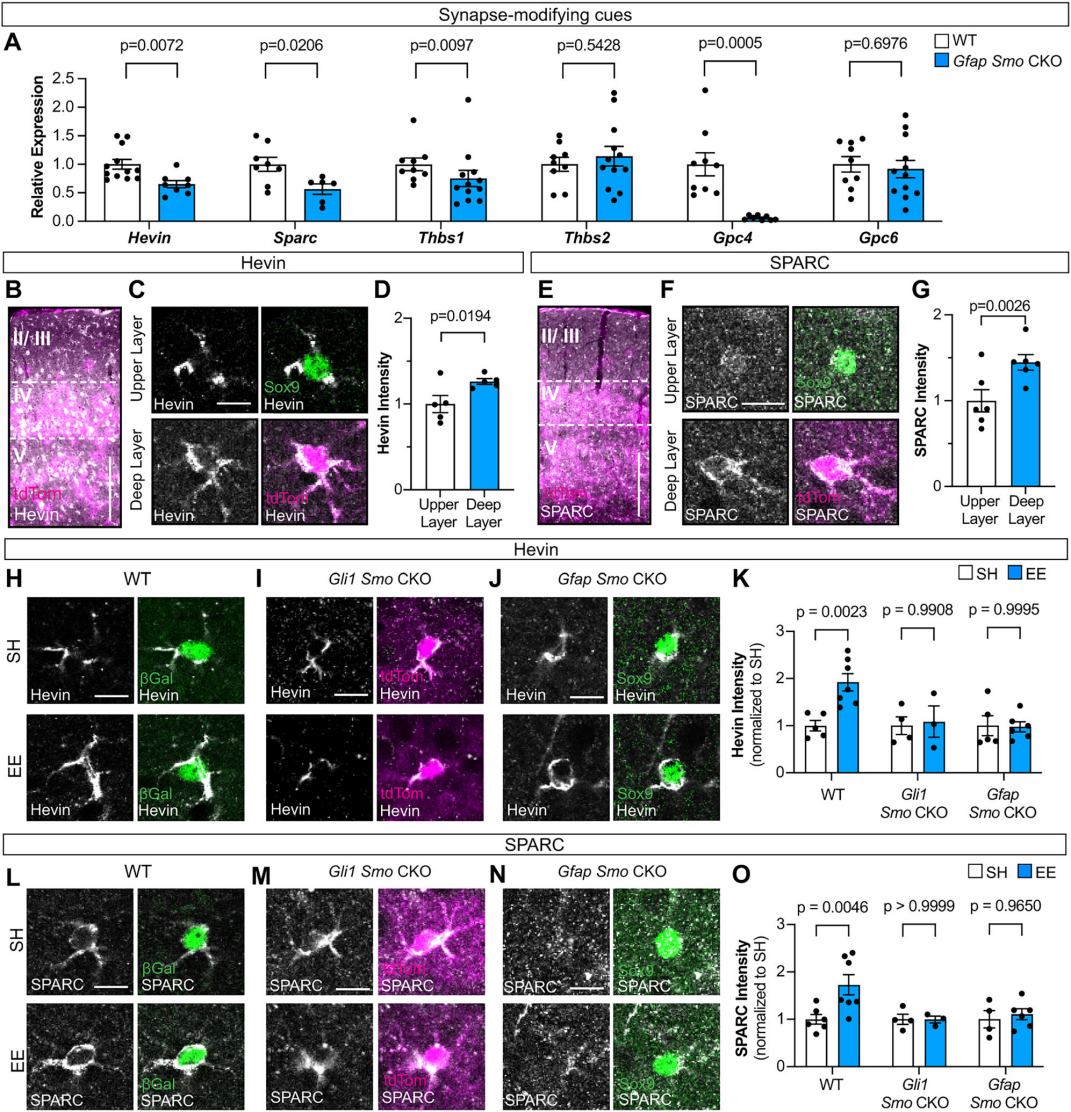
Shh regulates synapse-modifying cues Hevin and SPARC. ***A***, Relative expression measured by qRT-PCR of synapse-modifying genes *Hevin*, *Sparc*, *Thbs1*, *Thbs2*, *Gpc4*, and *Gpc6* (from left to right) in the cortex of adult WT versus *Gfap Smo* CKO mice, *n* = 6–12 animals per condition. Statistics: Student's *t* tests. ***B***, ***E***, Immunostaining for Hevin (gray, ***B***) and SPARC (gray, ***E***) in the cortex of P60 *Gli1^CreER/+^*;Ai14 mice showing tdTom cells (magenta) primarily localized to the deep layers (IV and V) of the cortex in contrast to upper layers (II/III). Scale bar, 250 μm. ***C***, ***F***, Immunofluorescent staining for Hevin (***C***, gray) and SPARC (***F***, gray) in individual astrocytes from upper layers labeled with Sox9 (green) and deep layers labeled with tdTom (magenta) in the cortex of P60 *Gli1^CreER/+^*;Ai14 mice. Merged images in the right panels. Scale bar, 10 μm. ***D***, ***G***, Analysis of fluorescent intensity of Hevin (***D***) and SPARC (***G***) immunostaining in individual astrocytes from the upper versus deep layers. *n* = 5 animals per condition, 25–50 cells analyzed per animal. Statistics: paired *t* tests. ***H***–***J***, ***L***–***N***, Immunofluorescent staining for Hevin (***H***–***J***) and SPARC (***L***–***N***; gray) in β-Gal cells (green) from *Gli1^nlacZ/+^* (WT) mice (***H***, ***L***), tdTom cells (magenta) from *Gli1 Smo* CKO mice (***I***, ***M***), and Sox9 cells (green) from *Gfap Smo* CKO mice (***J***, ***N***) housed in SH versus EE from P21 to P23. Merged images in the right panels. Scale bar, 10 μm. ***K***, ***O***, Analysis of Hevin (***K***) and SPARC (***O***) fluorescent intensity in individual astrocytes from WT (*n* = 5–7 mice), *Gli1 Smo* CKO (*n* = 3–4 mice), and *Gfap Smo* CKO (*n* = 5–6 mice) housed in SH versus EE. Statistics: two-way ANOVA with Sidak's multiple comparisons. In all graphs, data points represent individual animals, bars show mean ± SEM.

In the developing neural tube, Shh acts on neural progenitor cells to regulate the expression of genes that drive morphogenesis and cell-type specification. In astrocytes, several lines of evidence demonstrate that Shh regulates genes associated with synapse formation and function, including *Sparc*, *Kcnj10*, and *Gria1* (Farmer et al., 2016; Hill et al., 2019; Xie et al., 2022). To identify Shh-dependent genes mediating experience-dependent synaptic plasticity, we measured the gene expression of several established astrocyte-derived proteins that regulate synapses (Allen and Eroglu, 2017). We measured the expression of *Hevin*, *Sparc*, *Thbs1*, *Thbs2*, *Gpc4*, and *Gpc6* by qRT-PCR from whole cortical lysates of WT and *Gfap Smo* CKO mice. Interestingly, only *Thbs2* and *Gpc6* showed no difference between WT and *Gfap Smo* CKO mice (Fig. 9*A*), demonstrating a central role for Shh signaling in regulating expression of several astrocyte genes associated with synapse modification. We found an almost complete ablation of *Gpc4* in the cortex of *Gfap Smo* CKO mice, consistent with a role for *Gpc4* in regulating GluA1 at synapses (Allen et al., 2012; Fig. 9*A*).

*Hevin* and *Sparc* encode structurally related matricellular proteins belonging to the secreted protein acidic and rich in cysteine (SPARC) family (Kucukdereli et al., 2011; Fan et al., 2021). Both genes act to modify synapses structurally and functionally. *Hevin* induces structural synapse formation and is required for the maturation of thalamocortical synapses in layer IV (Risher et al., 2014) whereas *Sparc* has been shown to regulate GluA1 expression (Jones et al., 2018). Further, *Sparc* expression is upregulated in *Glast Ptch1* CKO mice in which Shh signaling is elevated in astrocytes, demonstrating a role for Shh in its regulation (Xie et al., 2022). We therefore chose to focus on these genes for further analysis.

To examine whether experience-dependent Shh activity regulates *Hevin*, we examined its expression in the cortex of *Gli1^CreER/+^*;Ai14 mice. Hevin immunostaining in the cortex shows an enrichment in layers IV and V, where Shh activity is highest (Fig. 9*B*). Single-cell analysis of deep- and upper-layer astrocytes in *Gli1^CreER/+^*;Ai14 mice revealed that astrocytes in layers IV and V, identified by tdTom labeling, express higher levels of Hevin compared with those in layers II/III, identified by Sox9 alone (Fig. 9*C*,*D*), consistent with a role for Shh signaling in regulating its expression. We next examined SPARC expression, which showed a similar enrichment in deep-layer, relative to upper-layer, astrocytes in WT mice (Fig. 9*E*–*G*). qRT-PCR analysis of *Hevin* and *SPARC* in *Gfap Smo* CKO mice showed a significant reduction in expression compared with WT controls (Fig. 9*A*). Neither *Hevin* nor *Sparc* expression was completely abolished in *Gfap Smo* CKO mice, suggesting that Shh signaling may cooperate with additional mechanisms to regulate their expression. Although their expression is higher in the deep layers of the cortex, their expression is not absent in the upper layers (Fig. 9*B*–*G*), suggesting differential regulation of these genes in specific astrocyte populations. Taken together, these data show that *Hevin* and *Sparc* are enriched in deep-layer astrocytes and further demonstrate that Shh signaling regulates their expression.

We next asked whether experience-dependent Shh activity promotes an increase in expression of these synapse-modifying cues. We performed immunostaining for Hevin and SPARC in *Gli1^nlacZ/+^* mice housed in SH or EE from P21 to P23 and analyzed their expression selectively in β-Gal+ cells in the deep layers of the cortex. Mice housed in EE showed a significant increase in Hevin and SPARC compared with SH controls (Fig. 9*H*,*K*,*L*,*O*), demonstrating that enriched sensory experience promotes upregulation of these proteins. Notably, exposure to EE failed to increase Hevin or SPARC in both *Gli1 Smo* CKO (Fig. 9*I*,*K*,*M*,*O*) and *Gfap Smo* CKO mice (Fig. 9*J*,*K*,*N*,*O*), demonstrating a requirement for Shh signaling in experience-dependent upregulation of these genes.

## Discussion

In this study, we demonstrate that neural activity stimulates Shh signaling between neurons and astrocytes. The activity of the pathway is highly dynamic and bidirectionally regulated by neural activity, key features for effective translation of experience into functional gene expression. Neural activity also promotes upregulation of Hevin and SPARC in a Shh-dependent manner. Selective perturbation of the pathway in astrocytes occludes experience-dependent synaptic plasticity in deep-layer, but not upper-layer, cortical synapses, demonstrating highly localized regulation over astrocyte modulation of synapses. Notably, a recent, unbiased screen for transcription factors expressed in response to neuronal activation also identified *Gli1* (Sardar et al., 2023). Taken together, these data demonstrate that neural activity stimulates Shh signaling and identify the Shh pathway as a molecular mechanism by which astrocytes couple neuronal activity to gene expression, promoting synaptic plasticity.

In addition to gene expression, activity also stimulates morphological plasticity of astrocytes that may promote synapse stabilization (Genoud et al., 2006; Bernardinelli et al., 2014). We did not observe changes in gross astrocyte morphology in response to sensory experience using our whisker stimulation protocol. It should be noted that our analyses relied on light microscopy and do not rule out increased astrocyte contact with synapses that are best observed by EM or increased dynamic interactions between astrocytes and synapses that require live imaging. Because our experiments were performed in juvenile mice, a period during which astrocytes undergo activity-dependent developmental maturation (Morel et al., 2014; Stogsdill et al., 2017; Cheng et al., 2023), we also cannot rule out a role for development. Astrocyte morphogenesis is regulated, in part, by brain-derived neurotrophic factor (BDNF) signaling which is secreted in an activity-dependent manner (Holt et al., 2019). Indeed, there is evidence that noncanonical Shh signaling in neurons interacts with BDNF in the developing hippocampus (Delmotte et al., 2020). However, whether there is cross talk between *Gli1*-mediated canonical Shh signaling in astrocytes and BDNF signaling is not known. Furthermore, whether activity-dependent Shh signaling in astrocytes is restricted to young mice or whether this mechanism can be leveraged in adult or aged mice to promote synaptic plasticity remains an open question.

While experience increases the number of astrocytes transducing SHH, experience has no effect on *Shh* expression in neurons. This suggests that Shh signaling is initiated by a defined population of neurons that can modulate the number of astrocytes they communicate with depending on need. In this way, Shh signaling cooperates with glutamatergic signaling, acting as a molecular mechanism by which neurons recruit astrocytes as needed to promote synaptic interactions. Precisely how SHH is released from neurons is poorly understood. Several lines of evidence point to axonal trafficking and release (Wallace and Raff, 1999; Traiffort et al., 2001), consistent with activity-dependent release. Studies in cell culture observed by EM demonstrate that SHH is localized with synaptic vesicles (Beug et al., 2011) and is released by high-frequency stimulation (Su et al., 2017). Although cells with *Gli1* activity are found most abundantly in layers IV and V of the cortex, we found few *Shh*-expressing neurons in the thalamus. Instead, *Shh* expression is found predominantly in layer V pyramidal neurons, suggesting that cortical Shh signaling is initiated by local neurons, rather than long-distance projections. This may arise from recurrent collateral innervation by layer IV and V neurons (Staiger and Petersen, 2020). Alternatively, SHH may be released by extracellular vesicles (Vyas et al., 2014; Coulter et al., 2018), which can be stimulated by neuronal activity (Sharma et al., 2013). The localization of *Gli1*-expressing cells in deep cortical layers, adjacent to *Shh*-expressing soma, is consistent with an exosomal source of SHH. These mechanisms are not mutually exclusive, and further work with SHH fusion proteins to identify their localization within specific cellular compartments could shed light on the precise mechanism by which neurons release SHH.

Our observation that Shh signaling can be regulated by neural activity uncovers a novel dimension to this versatile signaling pathway. Though best characterized for its roles as a morphogen and mitogen during embryonic neural development, several lines of evidence point to a role for SHH in regulating synapses. Shh signaling regulates expression of genes associated with glutamatergic signaling in Bergmann glia (Farmer et al., 2016) and modulation of Shh signaling in developing astrocytes regulates cortical synapses (Hill et al., 2019; Xie et al., 2022). The experiments in this study were performed on mice at P21, corresponding to the end of critical period plasticity for the somatosensory cortex (Pedrosa et al., 2022), suggesting that Shh signaling also mediates astrocyte modulation of mature, relatively established circuits. Collectively, these observations demonstrate that SHH promotes astrocyte modulation of synapses in both developing and mature circuits, adding to the broad repertoire of SHH-dependent cellular behaviors.

Here, we focus on Shh signaling in the somatosensory cortex, where its activity is localized primarily to deep-layer astrocytes. Our data show that genetic deletion of Shh signaling, selectively in layers IV and V, does not perturb structural plasticity in layers II/III, suggesting that astrocyte modulation of synapses is mediated by distinct mechanisms, even within a defined circuit. The requirement for Shh signaling at specific synapses is likely a function of its availability, rather than an intrinsic feature of specific microcircuits. Several components of the pathway, including *Gli2*, *Gli3*, and *Ptch1*, are found in upper-layer astrocytes (Garcia et al., 2010), suggesting these cells fail to receive sufficient ligand to initiate pathway activity. This suggests that promoting SHH availability to upper-layer astrocytes would promote similar effects on synapses. In support of this, ectopic expression of *Shh* in upper-layer neurons promotes expression of SHH target genes, including *Sparc* and *Kcnj10* (Xie et al., 2022). Thus, although Shh signaling is sufficient for regulating astrocyte gene expression and synapse remodeling, there is a differential requirement for the pathway among discrete astrocyte populations. The precise mechanism restricting SHH to deep layers is not known. Identifying these mechanisms, as well as those regulating experience-dependent gene expression in other astrocyte populations, will advance the discovery of strategies for promoting synaptic function and plasticity in injury or disease.

Astrocyte modification of synapses is mediated by a diverse repertoire of molecular cues, including the release and secretion of matricellular and cell adhesion molecules (Dallérac et al., 2018; Shan et al., 2021). Constitutive activation of Shh signaling in cortical astrocytes increases SPARC expression (Xie et al., 2022), suggesting SHH is sufficient for its upregulation. Here, we extend this finding and report that selective interruption of pathway activity in astrocytes significantly decreases SPARC expression, demonstrating a requirement for Shh signaling in SPARC expression. Hevin, a structurally related protein to SPARC, is also Shh-dependent (this study). We further demonstrate that both Hevin and SPARC show activity-dependent increases in expression that are occluded in *Gli1 Smo* CKO mice. Hevin promotes the structural formation of synapses through its interactions with neurexin and neuroligin and also promotes spine maturation (Risher et al., 2014; Singh et al., 2016). Our data show that both spine density and the proportion of spines with a mushroom morphology increase in a SHH-dependent manner following sensory experience, consistent with the idea that experience-dependent structural plasticity is mediated by upregulation of Hevin (Risher et al., 2014). In contrast to Hevin, the actions of SPARC on synapses are less well understood. SPARC has been shown to both antagonize and promote synapses (Kucukdereli et al., 2011; Jones et al., 2018). Our data showing that synapse density increases following sensory experience, despite an increase in SPARC expression, would seem to be in conflict with reports that SPARC antagonizes the synaptogenic actions of Hevin. An intriguing possibility is that Hevin and SPARC are differentially upregulated at specific synapses, enabling local regulation by specific perisynaptic astrocyte processes. In support of this, shRNA knockdown of SPARC in astrocytes selectively reduces corticocortical VGlut1-labeled synapses, but not thalamocortical VGlut2-labeled synapses in deep layers (Xie et al., 2022). Both the Hevin and SPARC antibodies used in this study were raised in the same species, precluding a direct test of this possibility. Future studies including EM approaches and rescue experiments are necessary to fully elucidate the precise roles of Hevin and SPARC in SHH-dependent astrocyte modulation of synapses.

Interestingly, we found that in addition to *Hevin* and *Sparc*, *Thbs1* and *Gpc4* are also SHH-dependent. Like Hevin, thrombospondins promote the structural formation of synapses (Christopherson et al., 2005), whereas glypican 4 promotes the recruitment of GluA1 AMPARs to the synapse (Allen et al., 2012). Thus, Shh signaling between neurons and astrocytes orchestrates the expression of several genes known to modify the structure and function of synapses. This suggests that SHH acts as a neuron-derived signal that recruits astrocytes during times of heightened activity to promote structural and functional modification of synapses. Whether these genes are direct or indirect targets of Shh signaling is unknown, and further studies are needed to elucidate the precise mechanism by which Shh signaling regulates such a broad array of synapse-modifying genes. An unbiased screen of Shh-dependent genes may reveal an even broader network of transcriptional programs underlying astrocyte regulation of synapses.

## References

[B1] Ahn S, Joyner AL (2005) In vivo analysis of quiescent adult neural stem cells responding to Sonic hedgehog. Nature 437:894–897. 10.1038/nature03994 16208373

[B2] Alexander GM, et al. (2009) Remote control of neuronal activity in transgenic mice expressing evolved G protein-coupled receptors. Neuron 63:27–39. 10.1016/j.neuron.2009.06.014 19607790 PMC2751885

[B3] Allen NJ, Bennett ML, Foo LC, Wang GX, Chakraborty C, Smith SJ, Barres BA (2012) Astrocyte glypicans 4 and 6 promote formation of excitatory synapses via GluA1 AMPA receptors. Nature 486:410–414. 10.1038/nature11059 22722203 PMC3383085

[B4] Allen NJ, Eroglu C (2017) Cell biology of astrocyte-synapse interactions. Neuron 96:697–708. 10.1016/j.neuron.2017.09.056 29096081 PMC5687890

[B5] Arellano J, Benavides-Piccione R, DeFelipe J, Yuste R (2007) Ultrastructure of dendritic spines: correlation between synaptic and spine morphologies. Front Neurosci 1:131–143. 10.3389/neuro.01.1.1.010.2007 18982124 PMC2518053

[B6] Armbruster BN, Li X, Pausch MH, Herlitze S, Roth BL (2007) Evolving the lock to fit the key to create a family of G protein-coupled receptors potently activated by an inert ligand. Proc Natl Acad Sci U S A 104:5163–5168. 10.1073/pnas.0700293104 17360345 PMC1829280

[B7] Bai CB, Auerbach W, Lee JS, Stephen D, Joyner AL (2002) Gli2, but not Gli1, is required for initial Shh signaling and ectopic activation of the Shh pathway. Development 129:4753–4761. 10.1242/dev.129.20.4753 12361967

[B8] Bernardinelli Y, et al. (2014) Activity-dependent structural plasticity of perisynaptic astrocytic domains promotes excitatory synapse stability. Curr Biol 24:1679–1688. 10.1016/j.cub.2014.06.025 25042585

[B9] Beug ST, Parks RJ, McBride HM, Wallace VA (2011) Processing-dependent trafficking of Sonic hedgehog to the regulated secretory pathway in neurons. Mol Cell Neurosci 46:583–596. 10.1016/j.mcn.2010.12.009 21182949

[B10] Blanco-Suarez E, Liu TF, Kopelevich A, Allen NJ (2018) Astrocyte-secreted chordin-like 1 drives synapse maturation and limits plasticity by increasing synaptic GluA2 AMPA receptors. Neuron 100:1116–1132.e13. 10.1016/j.neuron.2018.09.043 30344043 PMC6382071

[B11] Briscoe J, Therond PP (2013) The mechanisms of Hedgehog signalling and its roles in development and disease. Nat Rev Mol Cell Biol 14:416–429. 10.1038/nrm3598 23719536

[B12] Charytoniuk D, Porcel B, Rodriguez Gomez J, Faure H, Ruat M, Traiffort E (2002) Sonic Hedgehog signalling in the developing and adult brain. J Physiol Paris 96:9–16. 10.1016/S0928-4257(01)00075-4 11755778

[B13] Chen C-C, Bajnath A, Brumberg JC (2015) The impact of development and sensory deprivation on dendritic protrusions in the mouse barrel cortex. Cereb Cortex 25:1638–1653. 10.1093/cercor/bht415 24408954 PMC4506326

[B14] Cheng YT, Luna-Figueroa E, Woo J, Chen HC, Lee ZF, Harmanci AS, Deneen B (2023) Inhibitory input directs astrocyte morphogenesis through glial GABA(B)R. Nature 617:369–376. 10.1038/s41586-023-06010-x 37100909 PMC10733939

[B15] Christopherson KS, Ullian EM, Stokes CC, Mullowney CE, Hell JW, Agah A, Lawler J, Mosher DF, Bornstein P, Barres BA (2005) Thrombospondins are astrocyte-secreted proteins that promote CNS synaptogenesis. Cell 120:421–433. 10.1016/j.cell.2004.12.020 15707899

[B16] Coulter ME, et al. (2018) The ESCRT-III protein CHMP1A mediates secretion of Sonic hedgehog on a distinctive subtype of extracellular vesicles. Cell Rep 24:973–986.e8. 10.1016/j.celrep.2018.06.100 30044992 PMC6178983

[B17] Dallérac G, Zapata J, Rouach N (2018) Versatile control of synaptic circuits by astrocytes: where, when and how? Nat Rev Neurosci 19:729–743. 10.1038/s41583-018-0080-6 30401802

[B18] Delmotte Q, Diabira D, Belaidouni Y, Hamze M, Kochmann M, Montheil A, Gaiarsa J-L, Porcher C, Belgacem YH (2020) Sonic hedgehog signaling agonist (SAG) triggers BDNF secretion and promotes the maturation of GABAergic networks in the postnatal rat hippocampus. Front Cell Neurosci 14:98. 10.3389/fncel.2020.00098 32425757 PMC7212340

[B19] Fan S, Gangwar SP, Machius M, Rudenko G (2021) Interplay between hevin, SPARC, and MDGAs: modulators of neurexin-neuroligin transsynaptic bridges. Structure 29:664–678.e6. 10.1016/j.str.2021.01.003 33535026 PMC8254758

[B20] Farhy-Tselnicker I, Boisvert MM, Liu H, Dowling C, Erikson GA, Blanco-Suarez E, Farhy C, Shokhirev MN, Ecker JR, Allen NJ (2021) Activity-dependent modulation of synapse-regulating genes in astrocytes. Elife 10:e70514. 10.7554/eLife.70514 34494546 PMC8497060

[B21] Farmer WT, et al. (2016) Neurons diversify astrocytes in the adult brain through sonic hedgehog signaling. Science 351:849–854. 10.1126/science.aab3103 26912893

[B22] Feng G, Mellor RH, Bernstein M, Keller-Peck C, Nguyen QT, Wallace M, Nerbonne JM, Lichtman JW, Sanes JR (2000) Imaging neuronal subsets in transgenic mice expressing multiple spectral variants of GFP. Neuron 28:41–51. 10.1016/S0896-6273(00)00084-2 11086982

[B23] Garcia AD, Doan NB, Imura T, Bush TG, Sofroniew MV (2004) GFAP-expressing progenitors are the principal source of constitutive neurogenesis in adult mouse forebrain. Nat Neurosci 7:1233–1241. 10.1038/nn1340 15494728

[B24] Garcia ADR, Petrova R, Eng L, Joyner AL (2010) Sonic hedgehog regulates discrete populations of astrocytes in the adult mouse forebrain. J Neurosci 30:13597–13608. 10.1523/JNEUROSCI.0830-10.2010 20943901 PMC2966838

[B25] Genoud C, Quairiaux C, Steiner P, Hirling H, Welker E, Knott GW (2006) Plasticity of astrocytic coverage and glutamate transporter expression in adult mouse cortex. PLoS Biol 4:e343. 10.1371/journal.pbio.0040343 17048987 PMC1609127

[B26] Gingrich EC, Case K, Garcia ADR (2022) A subpopulation of astrocyte progenitors defined by Sonic hedgehog signaling. Neural Dev 17:2. 10.1186/s13064-021-00158-w 35027088 PMC8759290

[B27] Graves AR, et al. (2021) Visualizing synaptic plasticity in vivo by large-scale imaging of endogenous AMPA receptors. Elife 10:e66809. 10.7554/eLife.66809 34658338 PMC8616579

[B28] Harfe BD, Scherz PJ, Nissim S, Tian H, McMahon AP, Tabin CJ (2004) Evidence for an expansion-based temporal Shh gradient in specifying vertebrate digit identities. Cell 118:517–528. 10.1016/j.cell.2004.07.024 15315763

[B29] Harwell CC, Parker PR, Gee SM, Okada A, McConnell SK, Kreitzer AC, Kriegstein AR (2012) Sonic hedgehog expression in corticofugal projection neurons directs cortical microcircuit formation. Neuron 73:1116–1126. 10.1016/j.neuron.2012.02.009 22445340 PMC3551478

[B30] Hasel P, et al. (2017) Neurons and neuronal activity control gene expression in astrocytes to regulate their development and metabolism. Nat Commun 8:1–17. 10.1038/ncomms15132 28462931 PMC5418577

[B31] Hill SA, Blaeser AS, Coley AA, Xie Y, Shepard KA, Harwell CC, Gao W-J, Garcia ADR (2019) Sonic hedgehog signaling in astrocytes mediates cell type-specific synaptic organization. Elife 8:e45545. 10.7554/eLife.45545 31194676 PMC6629371

[B32] Holt LM, Hernandez RD, Pacheco NL, Torres Ceja B, Hossain M, Olsen ML (2019) Astrocyte morphogenesis is dependent on BDNF signaling via astrocytic TrkB.T1. Elife 8:e44667. 10.7554/eLife.44667 31433295 PMC6726422

[B33] Holtmaat A, Svoboda K (2009) Experience-dependent structural synaptic plasticity in the mammalian brain. Nat Rev Neurosci 10:647–658. 10.1038/nrn2699 19693029

[B34] Hrvatin S, et al. (2018) Single-cell analysis of experience-dependent transcriptomic states in the mouse visual cortex. Nat Neurosci 21:120–129. 10.1038/s41593-017-0029-5 29230054 PMC5742025

[B35] Hui C-C, Angers S (2011) Gli proteins in development and disease. Annu Rev Cell Dev Biol 27:513–537. 10.1146/annurev-cellbio-092910-154048 21801010

[B36] Jones EV, Bernardinelli Y, Zarruk JG, Chierzi S, Murai KK (2018) SPARC and GluA1-containing AMPA receptors promote neuronal health following CNS injury. Front Cell Neurosci 12:22. 10.3389/fncel.2018.00022 29449802 PMC5799273

[B37] Jung CKE, Herms J (2014) Structural dynamics of dendritic spines are influenced by an environmental enrichment: an in vivo imaging study. Cereb Cortex 24:377–384. 10.1093/cercor/bhs317 23081882

[B38] Kucukdereli H, et al. (2011) Control of excitatory CNS synaptogenesis by astrocyte-secreted proteins Hevin and SPARC. Proc Natl Acad Sci U S A 108:E440–E449. 10.1073/pnas.1104977108 21788491 PMC3156217

[B39] Landers MS, Knott GW, Lipp HP, Poletaeva I, Welker E (2011) Synapse formation in adult barrel cortex following naturalistic environmental enrichment. Neuroscience 199:143–152. 10.1016/j.neuroscience.2011.10.040 22061424

[B40] Long F, Zhang XM, Karp S, Yang Y, McMahon AP (2001) Genetic manipulation of hedgehog signaling in the endochondral skeleton reveals a direct role in the regulation of chondrocyte proliferation. Development 128:5099–5108. 10.1242/dev.128.24.5099 11748145

[B41] Madisen L, et al. (2009) A robust and high-throughput Cre reporting and characterization system for the whole mouse brain. Nat Neurosci 13:133–140. 10.1038/nn.2467 20023653 PMC2840225

[B42] Makino H, Malinow R (2011) Compartmentalized versus global synaptic plasticity on dendrites controlled by experience. Neuron 72:1001–1011. 10.1016/j.neuron.2011.09.036 22196335 PMC3310180

[B43] Matsuzaki M, Honkura N, Ellis-Davies GCR, Kasai H (2004) Structural basis of long-term potentiation in single dendritic spines. Nature 429:761–766. 10.1038/nature02617 15190253 PMC4158816

[B44] Morel L, Higashimori H, Tolman M, Yang Y (2014) VGluT1+ neuronal glutamatergic signaling regulates postnatal developmental maturation of cortical protoplasmic astroglia. J Neurosci 34:10950–10962. 10.1523/JNEUROSCI.1167-14.2014 25122895 PMC4131010

[B45] Nagai J, Rajbhandari AK, Gangwani MR, Hachisuka A, Coppola G, Masmanidis SC, Fanselow MS, Khakh BS (2019) Hyperactivity with disrupted attention by activation of an astrocyte synaptogenic Cue. Cell 177:1280–1292.e20. 10.1016/j.cell.2019.03.019 31031006 PMC6526045

[B46] Niewiadomski P, Niedziólka SM, Markiewicz L, Uspienski T, Baran B, Chojnowska K (2019) Gli proteins: regulation in development and cancer. Cells 8:147. 10.3390/cells8020147 30754706 PMC6406693

[B47] Nimchinsky EA, Sabatini BL, Svoboda K (2002) Structure and function of dendritic spines. Annu Rev Physiol 64:313–353. 10.1146/annurev.physiol.64.081501.160008 11826272

[B48] Pedrosa LRR, Coimbra G, Corrêa MG, Dias IA, Bahia CP (2022) Time window of the critical period for neuroplasticity in S1, V1, and A1 sensory areas of small rodents: a systematic review. Front Neuroanat 16:763245. 10.3389/fnana.2022.763245 35370567 PMC8970055

[B49] Peng J, Fabre PJ, Dolique T, Swikert SM, Kermasson L, Shimogori T, Charron F (2018) Sonic hedgehog is a remotely produced cue that controls axon guidance trans-axonally at a midline choice point. Neuron 97:326–340.e4. 10.1016/j.neuron.2017.12.028 29346753

[B50] Risher WC, et al. (2014) Astrocytes refine cortical connectivity at dendritic spines. Elife 3:e04047. 10.7554/eLife.04047 25517933 PMC4286724

[B51] Sardar D, et al. (2023) Induction of astrocytic Slc22a3 regulates sensory processing through histone serotonylation. Science 380:eade0027. 10.1126/science.ade0027 37319217 PMC10874521

[B52] Shan L, Zhang T, Fan K, Cai W, Liu H (2021) Astrocyte-neuron signaling in synaptogenesis. Front Cell Dev Biol 9:680301. 10.3389/fcell.2021.680301 34277621 PMC8284252

[B53] Sharma P, Schiapparelli L, Cline HT (2013) Exosomes function in cell-cell communication during brain circuit development. Curr Opin Neurobiol 23:997–1004. 10.1016/j.conb.2013.08.005 23998929 PMC3830597

[B54] Singh SK, et al. (2016) Astrocytes assemble thalamocortical synapses by bridging NRX1α and NL1 via Hevin. Cell 164:183–196. 10.1016/j.cell.2015.11.034 26771491 PMC4715262

[B55] Staiger JF, Petersen CCH (2020) Neuronal circuits in barrel cortex for whisker sensory perception. Physiol Rev 101:353–415. 10.1152/physrev.00019.2019 32816652

[B56] Stogsdill JA, Ramirez J, Liu D, Kim YH, Baldwin KT, Enustun E, Ejikeme T, Ji RR, Eroglu C (2017) Astrocytic neuroligins control astrocyte morphogenesis and synaptogenesis. Nature 551:192–197. 10.1038/nature24638 29120426 PMC5796651

[B57] Su Y, Yuan Y, Feng S, Ma S, Wang Y (2017) High frequency stimulation induces sonic hedgehog release from hippocampal neurons. Sci Rep 7:43865. 10.1038/srep43865 28262835 PMC5338313

[B58] Sun W, et al. (2017) SOX9 is an astrocyte-specific nuclear marker in the adult brain outside the neurogenic regions. J Neurosci 37:4493–4507. 10.1523/JNEUROSCI.3199-16.2017 28336567 PMC5413187

[B59] Takahashi T, Svoboda K, Malinow R (2003) Experience strengthening transmission by driving AMPA receptors into synapses. Science 299:1585–1588. 10.1126/science.1079886 12624270

[B60] Tan HL, Roth RH, Graves AR, Cudmore RH, Huganir RL (2020) Lamina-specific AMPA receptor dynamics following visual deprivation in vivo. Elife 9:e52420. 10.7554/eLife.52420 32125273 PMC7053996

[B61] Traiffort E, Charytoniuk DA, Faure H, Ruat M (1998) Regional distribution of Sonic Hedgehog, patched, and smoothened mRNA in the adult rat brain. J Neurochem 70:1327–1330. 10.1046/j.1471-4159.1998.70031327.x 9489757

[B62] Traiffort E, Charytoniuk D, Watroba L, Faure H, Sales N, Ruat M (1999) Discrete localizations of hedgehog signalling components in the developing and adult rat nervous system. Eur J Neurosci 11:3199–3214. 10.1046/j.1460-9568.1999.00777.x 10510184

[B63] Traiffort E, Moya KL, Faure H, Hässig R, Ruat M (2001) High expression and anterograde axonal transport of aminoterminal sonic hedgehog in the adult hamster brain. Eur J Neurosci 14:839–850. 10.1046/j.0953-816x.2001.01708.x 11576188

[B64] Vyas N, Walvekar A, Tate D, Lakshmanan V, Bansal D, Lo Cicero A, Raposo G, Palakodeti D, Dhawan J (2014) Vertebrate Hedgehog is secreted on two types of extracellular vesicles with different signaling properties. Sci Rep 4:7357. 10.1038/srep07357 25483805 PMC4258658

[B65] Wallace VA, Raff MC (1999) A role for Sonic hedgehog in axon-to-astrocyte signalling in the rodent optic nerve. Development 126:2901–2909. 10.1242/dev.126.13.2901 10357934

[B66] Xie Y, et al. (2022) Astrocyte-neuron crosstalk through Hedgehog signaling mediates cortical synapse development. Cell Rep 38:110416. 10.1016/j.celrep.2022.110416 35196485 PMC8962654

[B67] Yang G, Pan F, Gan W-B (2009) Stably maintained dendritic spines are associated with lifelong memories. Nature 462:920–924. 10.1038/nature08577 19946265 PMC4724802

[B68] Yap EL, Greenberg ME (2018) Activity-regulated transcription: bridging the gap between neural activity and behavior. Neuron 100:330–348. 10.1016/j.neuron.2018.10.013 30359600 PMC6223657

[B69] Zhang Y, Cudmore RH, Lin D-T, Linden DJ, Huganir RL (2015) Visualization of NMDA receptor–dependent AMPA receptor synaptic plasticity in vivo. Nat Neurosci 18:402–407. 10.1038/nn.3936 25643295 PMC4339371

[B70] Zuo Y, Yang G, Kwon E, Gan W-B (2005) Long-term sensory deprivation prevents dendritic spine loss in primary somatosensory cortex. Nature 436:261–265. 10.1038/nature03715 16015331

