# Supplementary figures and images for: Astrocyte Modulation of Synaptic Plasticity Mediated by Activity-Dependent Sonic Hedgehog Signaling
Source: J Neurosci. 2025 Feb 3;45(11):e1336242025. doi: 10.1523/JNEUROSCI.1336-24.2025 (PMC11905353; doi:10.1523/JNEUROSCI.1336-24.2025)

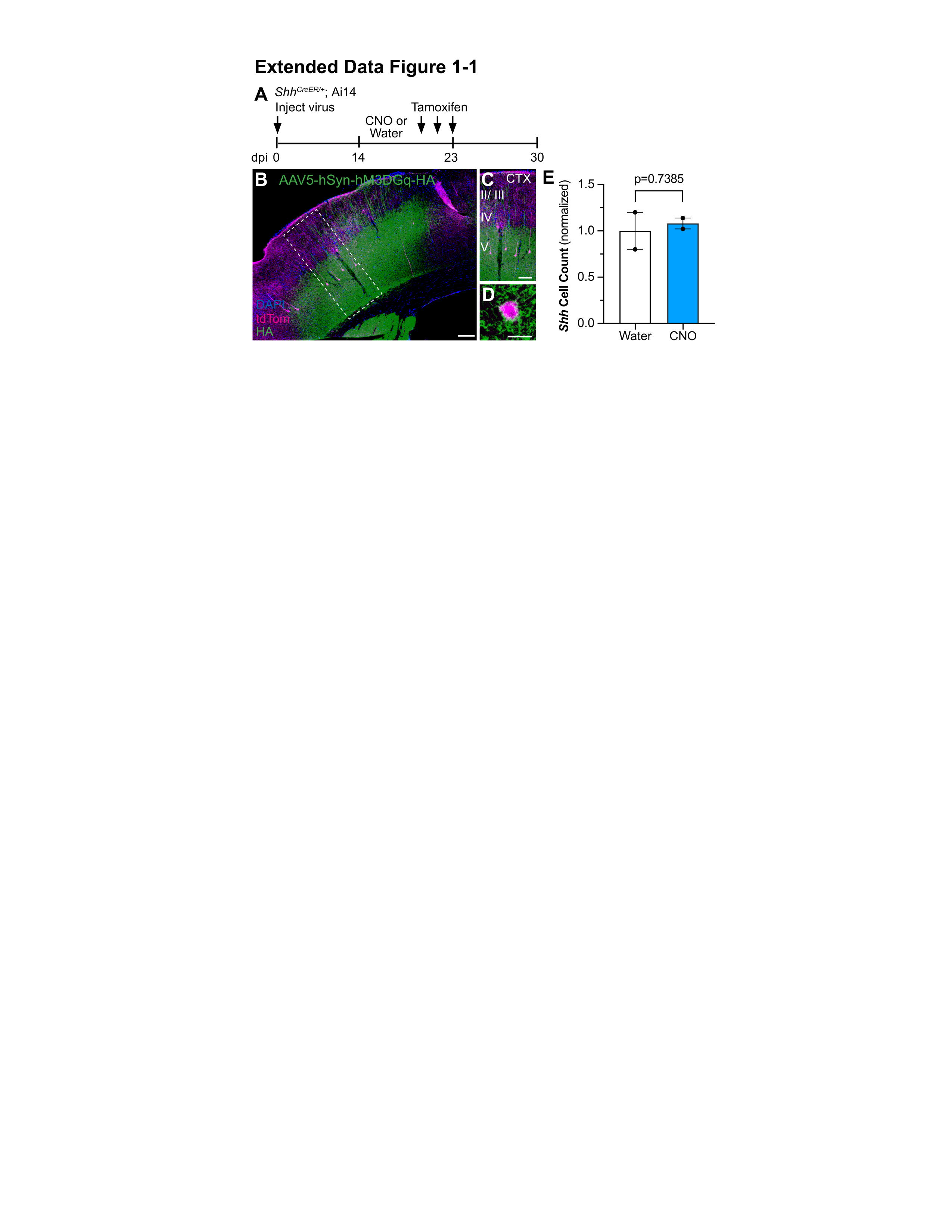

Supplement: Figure 1-1 — Chemogenetic stimulation of neuronal activity does not increase the number of Shh-expressing neurons. (A) Schematic depicting experimental approach to determine whether chemogenetic stimulation of neuronal activity increases the number of Shh-expressing cells. ShhCreER/+; Ai14 mice were injected with AAV5-hSyn-hM3DGq-HA targeting the somatosensory cortex. After 2 weeks, mice were fed CNO-medicated or unmedicated water for 10 days, receiving 3 doses of tamoxifen during the last 3 days. Tissues were analyzed seven days later. dpi, days post injection. (B-D) Immunolabeling for HA (green) shows the transduced region overlapping with tdTom labeled (magenta) cells in the cortex. Inset shown in (C). Individual cell shown at high power in (D). Counterstained with DAPI (blue). Scale bar, 25 μm. (E) The number of Shh neurons in the cortex between water control versus CNO. Data points represent individual animals, n = 2 mice in water group, n = 2 mice in CNO group. Bars show mean ± SEM. Statistic: Student’s t-test. Download Figure 1-1, TIF file. [file jneuro-45-e1336242025-s001.tif]

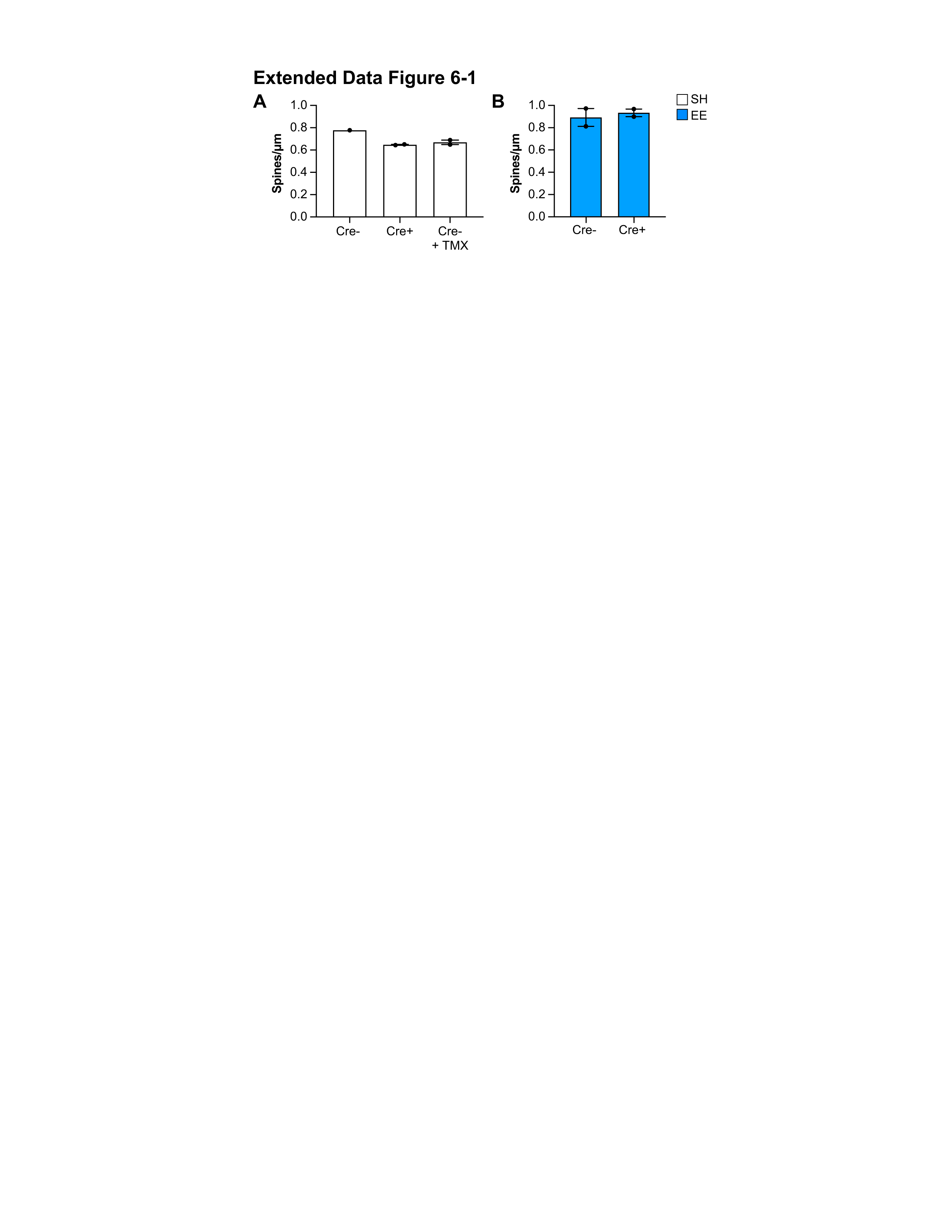

Supplement: Figure 6-1 — Wild-type littermate controls for comparison with Gli1 Smo CKO were pooled from different conditions. (A) Spine density of deep layer dendritic segments in wild-type littermate controls of Gli1 Smo CKO animals housed in SH, including Cre- mice with and without tamoxifen and Cre + mice without tamoxifen. Animals were subsequently pooled as WT for comparison with Gli1 Smo CKO. (B) Spine density of deep layer dendritic segments in wild-type littermate controls of Gli1 Smo CKO animals housed in EE, including Cre- and Cre + without tamoxifen. Animals were subsequently pooled as WT for comparison with Gli1 Smo CKO. Data points represent individual animals; bars show mean ± SEM. Download Figure 6-1, TIF file. [file jneuro-45-e1336242025-s002.tif]
